# Pan-Cancer Analyses Reveal Oncogenic Role and Prognostic Value of F-Box Only Protein 22

**DOI:** 10.3389/fonc.2021.790912

**Published:** 2022-01-24

**Authors:** Sen Chen, Shuangxin Ma, Jiaoyan Yan, Haiqing Wang, Bojiao Ding, Zihu Guo, Yaohua Ma, Xuetong Chen, Yonghua Wang

**Affiliations:** ^1^ Center of Bioinformatics, College of Life Science, Northwest A & F University, Yangling, China; ^2^ Department of Basic Clinical Laboratory Medicine, School of Clinical Laboratory Science, Guizhou Medical University, Guiyang, China; ^3^ Key Laboratory of Resource Biology and Biotechnology in Western China, Ministry of Education, School of Life Sciences, Northwest University, Xi’an, China

**Keywords:** pan-cancer, FBXO22, ubiquitination, cell cycle, F-box only protein 22

## Abstract

The F-box protein 22 (FBXO22), an F-box E3 ligase, has been identified to be critically involved in carcinogenesis. However, a systematic assessment of the role of FBXO22 across human cancers is lacking. Here, we performed a pan-cancer analysis to explore the role of FBXO22 in 33 cancer types using multiomic data from The Cancer Genome Atlas (TCGA). First, we found that high FBXO22 expression in multiple cancers was closely associated with poor overall survival and relapse-free survival. Next, we identified ten proteins that interact with FBXO22 and 13 of its target substrates using the STRING database and a literature search to explore the regulatory role of FBXO22 in tumorigenesis. Genes encoding these proteins were found to be significantly enriched in cell cycle negative regulation and ubiquitination pathways. This was confirmed in nonsmall cell lung cancer A549 cells, where FBXO22 overexpression enhanced cyclin-dependent kinase 4 (CDK4) protein levels and promoted cell proliferation. Similarly, overexpression or interference of FBXO22 changed the protein level of one of its substrates, PTEN. Additionally, we found that FBXO22 mutations were accompanied by altered substrate expression, especially in uterine corpus endometrial carcinoma and lung adenocarcinoma; endometrial carcinoma patients with FBXO22 genetic alterations also had better overall and relapse-free survival. Notably, FBXO22 methylation levels were also decreased in most tumors, and hypomethylation of FBXO22 was associated with poor overall survival, relapse-free interval, and progression-free interval in pancreatic adenocarcinoma. Finally, we analyzed the correlation between the abundance of tumor infiltrating lymphocytes (TILs) and FBXO22 expression, copy number variation, and methylation. Multiple algorithms revealed that high FBXO22 expression was associated with lower TIL levels, especially in lung adenocarcinoma, lung squamous cell carcinoma, and sarcoma. Taken together, our findings demonstrate that FBXO22 degrades tumor suppressor genes by ubiquitination and inhibits the cell cycle to promote nonsmall cell lung cancer progression. Our study also provides a relatively comprehensive understanding of the oncogenic role of FBXO22 in different tumors.

## 1 Introduction

The correlations between protein posttranslational modifications (PTMs) and cancer progression have been extensively studied. Targeting regulators of PTM represents a promising strategy for anticancer treatment. Thus, mining cancer genomic data from The Cancer Genome Atlas (TCGA) and Gene Expression Omnibus (GEO) databases to explore the clinical prognosis and potential molecular mechanisms of PTM-related genes is important.

F-Box only protein 22 (FBXO22), a PTM regulator, is a member of the F-box protein family with E3 ligase activity (1). Recently, FBXO22 has shown to mediate ubiquitination of multiple proteins and has been linked to tumorigenesis (2). FBXO22 is involved in cell development and differentiation, including in cancer, by controlling the stability of lysine demethylase 4A (KDM4A) (3); FBXO22 ubiquitylates p53 and forms a complex with KDM4A to regulate cellular senescence (4). Lysine demethylase 4B (KDM4B) degradation in breast cancer cells is also mediated by FBXO22, leading to modulation of selective estrogen receptor modulator (SERM) activity and thus tamoxifen resistance in estrogen receptor (ER)-positive breast cancer cells (5). FBXO22 has also been shown to target MDM2 proto-oncogene (HDM2) for ubiquitination and degradation, thereby inhibiting breast cancer invasion and metastasis (6). One study showed that a patient-derived tryptophan-to-arginine mutation at residue 52 (W52R) within the F-box domain impaired FBXO22 binding to the SKP1–Cullin1 complex, thus blocking FBXO22-mediated snail family transcriptional repressor 1 (SNAIL) degradation and abrogating FBXO22 suppression of breast cancer cell migration, invasion, and metastasis (7). These findings suggest that FBXO22 plays dual roles in promoting proliferation and suppressing metastasis in breast cancer.

FBXO22 also enhances the ubiquitylation of p21 (1) and KLF4 (8) to promote hepatocellular carcinoma progression. FBXO22 can reverse cisplatin resistance in tumor cells by mediating polyubiquitination and degradation of basigin (BSG, also known as CD147) through its recognition of the BSG intracellular domain (9). FBXO22 mediates lys-63-linked liver kinase B1 (LKB1) polyubiquitination and inhibits its kinase activity, thereby inhibiting nonsmall-cell lung cancer (NSCLC) cell growth (10). In addition, FBXO22 was shown to mediate BTB domain and CNC homolog 1 (BACH1) degradation, inhibiting migration in lung cancer cells (11). It was reported that FBXO22 ubiquitinates and degrades nuclear phosphatase and tensin homolog (PTEN) *via* proteasome-mediated degradation in colorectal cancer, leading to tumorigenesis (12).

One study revealed a mechanism by which FBXO22 recognizes the motif XXPpSPXPXX as a conserved phosphodegron to target substrates for destruction and demonstrated that FBXO22 mediates BAG cochaperone 3 (BAG3) ubiquitination and degradation, which is involved in tumorigenesis (13). A recent study also found that FBXO22 degraded PH domain and leucine-rich repeat protein phosphatase 1 (PHLPP1) by ubiquitination, thus ameliorating rotenone-induced neurotoxicity (14), but the interaction of FBXO22 with PHLPP1 in tumors is unknown.

In addition to targeting substrates for ubiquitination for degradation, recent studies have identified FBXO22 as a regulator of hypoxia-inducible factor 1 subunit alpha (HIF1α), vascular endothelial growth factor A (VEGFA), matrix metalloproteinase-1 (TIMP-1), and matrix metalloproteinase-9 (MMP-9) (15, 16). FBXO22 promotes melanoma cell motility and angiogenesis *via* upregulating HIF1α and VEGFA (15). FBXO22 was previously shown to have no effect on renal cell carcinoma (RCC) cell proliferation, but FBXO22 was shown to limit RCC cell motility and reverse epithelial-to-mesenchymal transition (EMT) by increasing TIMP-1 activity, decreasing MMP-9 expression, and reducing VEGF secretion (16).

These studies suggest that FBXO22 plays an important role in tumorigenesis, especially in mediating ubiquitinated degradation of proteins. However, the role of FBXO22-mediated ubiquitination of substrates in human cancer is poorly understood. Here, we performed a pan-cancer analysis of FBXO22 using TCGA database to explore the role of FBXO22-mediated ubiquitination in cancer. We also analyzed a group of factors, such as gene expression, survival status, DNA methylation, genetic alteration, immune infiltration, and relevant cellular pathway, to investigate the potential molecular mechanism of FBXO22 in the pathogenesis or clinical prognosis of different cancers.

## 2 Materials and Methods

### 2.1 Gene Expression Analysis

First, we used FBXO22 as a query in the “Tissue Atlas” module of The Human Protein Atlas web service (www.proteinatlas.org) in order to obtain FBXO22 expression levels in different healthy cell and tissue types. Next, we downloaded HTSeq-FPKM data for 33 cancer types from TCGA using the software package TCGAbiolinks (v2.20.0) (17) in R (v4.1.0) to explore FBXO22 expression levels in different cancers. For cancer types containing a number of healthy samples greater than ten, including bladder urothelial carcinoma (BLCA), breast invasive carcinoma (BRCA), colon adenocarcinoma (COAD), esophageal carcinoma (ESCA), head and neck squamous cell carcinoma (HNSC), kidney chromophobe (KICH), kidney renal clear cell carcinoma (KIRC), kidney renal papillary cell carcinoma (KIRP), liver hepatocellular carcinoma (LIHC), lung adenocarcinoma (LUAD), lung squamous cell carcinoma (LUSC), prostate adenocarcinoma (PRAD), rectum adenocarcinoma (READ), stomach adenocarcinoma (STAD), thyroid carcinoma (THCA), and uterine corpus endometrial carcinoma (UCEC), we performed expression level analysis of the FBXO22 gene in healthy and tumor tissues using R. For cancer types with no healthy samples, or with a healthy sample number less than ten, including adrenocortical carcinoma (ACC), lymphoid neoplasm diffuse large B-cell lymphoma (DLBC), acute myeloid leukemia (LAML), brain lower-grade glioma (LGG), ovarian serous cystadenocarcinoma (OV), testicular germ cell tumors (TGCT), uterine carcinosarcoma (UCS), sarcoma (SARC), pancreatic adenocarcinoma (PAAD), glioblastoma multiforme (GBM), pheochromocytoma and paraganglioma (PCPG), thymoma (THYM), cholangiocarcinoma (CHOL), cervical squamous cell carcinoma and endocervical adenocarcinoma (CESC), and skin cutaneous melanoma (SKCM), we used the “Expression Analysis-Expression DIY-Box Plots” module of the GEPIA2 web server (18) to obtain box plots of the FBXO22 mRNA levels, as an indicator of gene expression, in tumor and healthy tissues by matching TCGA normal and genotype–tissue expression (GTEx) data. For an expression analysis of FBXO22 protein in BRCA, LUAD, and UCEC, we used data from the Clinical Proteomic Tumor Analysis Consortium (CPTAC) in the UALCAN web service (19, 20).

### 2.2 Survival and Prognosis Analysis

In the “Survival Analysis” module on the GEPIA2 web server (18), samples were stratified into high- and low-expression groups according to the median expression of *FBXO22* gene in each cancer type, and overall and relapse-free survival (RFS) analyses were performed. We performed a “tumor vs. normal” meta-analysis, a “survival” meta-analysis, and an “overall survival” (OS) analysis of *FBXO22* gene expression using the LUNG CANCER EXPLORER service (21). We used the Kaplan-Meier plotter to analyze the correlation between the FBXO22 expression and OS/RFS in pan-cancer (22).

### 2.3 Gene Enrichment Analysis

To explore the regulatory role of FBXO22 in tumorigenesis, we identified FBXO22-binding proteins using the STRING database (23) and collected FBXO22 substrates through a literature collection. Next, we performed Gene Ontology (GO) biological process enrichment analysis of genes encoding these proteins using the clusterProfiler package (v4.0.2) (24) in R.

### 2.4 Cell Lines and Culture

Human NSCLC cells (nci-h1975 and A549), human bronchial epithelial cells 16HBE, and HEK293T cells were purified from the Chinese Academy of Sciences Shanghai cell bank (Shanghai, China). A549 and 16HBE cells were cultured in Dulbecco’s modified Eagle’s medium (DMEM, Gibco, Waltham, MA, USA) supplemented with 10% fetal bovine serum (FBS, JRH Biosciences, Lenexa, KS, USA). Human NSCLC H1975 cells were cultured in RPMI-1640 medium (Gibco, Life Technologies, Waltham, CA, USA) with 10% FBS. For DNA methyltransferase inhibition assays, SGI-1027 (Shyuanye, Shanghai, China) was added to the culture medium for a final concentration of 2, 4, or 8 μM. All cells were cultured in a humidified cell incubator at 37°C with 5% CO_2_.

### 2.5 Cell Proliferation Experiments

Cell proliferation was evaluated using a Cell Counting Kit-8 (CCK8) assay (Best Bio, Shanghai, China). In brief, cells were seeded in 96-well plates at a concentration of 3 × 10^3^ per well and cultured for 1, 2, and 3 days. CCK-8 solution (10 µl) was added into each well at the indicated time points, then the plates were stored for 2 h at 37°C. Next, the number of viable cells was estimated by measuring the absorbance at 450 nm.

### 2.6 Western Blot Analysis

Cells were collected and lysed using Qproteome™ Mammalian Protein Prep Kit (Qiagen, Hilden, Germany). After centrifugation at 13,800×*g* for 10 min, the protein content in the supernatant was determined using the BCA protein assay kit (Bio-Rad, Shanghai, China). Equal amounts of protein were boiled by adding 4× sample loading buffers for 10 min at 100°C and resolved using sodium dodecylsulfate polyacrylamide gel electrophoresis (SDS-PAGE). Antibodies against α-tubulin (11224-1-AP), CDK4 (11026-1-AP), and FBXO22 (13606-1-AP) were purchased from Proteintech (Rosemont, IL, USA), DNTM1 (ab188453) were purchased from Abcam (Cambridge, MA, USA), and PTEN (9188S) and ubiquitin (3936S) were purchased from Cell Signaling Technology (Danvers, MA, USA).

### 2.7 Immunoprecipitation

Cells were harvested, lysed, and briefly sonicated. After centrifugation at 12,000×*g* for 10 min at 4°C, the supernatant (whole-cell lysate) was collected from each sample. PTEN antibodies were incubated with protein A magnetic beads at room temperature for 4 h, and unbound antibodies were washed off with elution buffer. Subsequently, the beads were incubated with cell lysate supernatants at 4°C overnight. The precipitates were washed three times with immunoprecipitation buffer, boiled in sample buffer, and Western blot analysis was then performed.

### 2.8 Plasmid Transfection and Lentivirus Packaging

Plasmids pLV[Exp]-EGFP:T2A:Puro-CMV>FLAG/hFBXO22[NM_147188.3] (VB210508-1064ndh), pRP[shRNA]-EGFP:P2A:Puro-U6>Scramble[shRNA#1] (VB200706-2709njm) and pRP[shRNA]-EGFP:P2A:Puro-U6>hFBXO22[shRNA#1] (VB210408-1046ugc) were purchased from VectorBuilder (Guangzhou, China). For the lentiviral packaging, we used pCMV delta R8.2 (Addgene 12263) and pCMV-VSV-G (Addgene 8454) systems. For plasmid transfection, we used Lipofectamine 2000 (Invitrogen, Waltham, MA, USA).

### 2.9 Genetic Alteration Analysis

The Catalogue of Somatic Mutations in Cancer (COSMIC) is the largest source of expert manually curated somatic mutation information relating to human cancers in the world (25). First, we used COSMIC to explore the distribution of different types of mutations in FBXO22. Next, we used the term “FBXO22” in the “Quick Search Beta” module of the cBioPortal web service (26, 27) to analyze the genetic alteration status of FBXO22 in different cancer types from TCGA cohorts. We then examined the association between genetic alterations in FBXO22 and clinical outcomes for UCEC. We used the UCEC (TCGA, PanCancer Atlas) dataset in our query. To explore the relationship between FBXO22 mutations and FBOXO22 substrate gene expression, we used the “Gene_Mutation” module in TIMER2 (28).

### 2.10 Methylation Analysis

To assess the methylation level of FBXO22 in cancer and healthy tissues, we used “TCGA” module in the UALCAN database to obtain boxplots for FBXO22 methylation level in COAD, PRAD, CESC, TGCT, READ, KIRP, LUAD, LUSC, BRCA, UCEC, and SARC (19). DNMIVD is a comprehensive annotation and interactive visualization database for DNA methylation profiles in diverse human cancers (29). The DNMIVD tool was used to investigate the correlation between expression, OS, disease-free interval, and FBXO22 promoter methylation levels. Searches were performed using FBXO22 as the input on the “Home” or “Model” page of the DNMIVD web service. Retrieved data and images were downloaded. The methylation sites cg08290738, cg00942495, and cg05374463 of FBXO22 were also used as inputs in other analyses.

### 2.11 Immune Cell Infiltration Analysis

TISIDB, a web portal for tumor and immune system interaction (30), was used to explore relationships between tumor-infiltrating lymphocyte (TILs) abundance and FBXO22 expression, copy number variation (CNV), and methylation. The activity of the tumor immunity cycle is a direct, integrated manifestation of the functions of the chemokine system and other immune regulators (31, 32). Thus, we also used TISIDB to analyze correlations between FBXO22 expression levels and chemokines, receptors, and three kinds of immunomodulators (immunoinhibitors, immunostimulators, and major histocompatibility complexes (MHCs)) across human cancers. For the relationship between the FBXO22 expression and the level of infiltrating natural killer (NK) T cells and myeloid-derived suppressor cells (MDSCs) across the diverse cancer types, we used the “Immune” module of the TIMER2 webserver (28). Data and pictures of the analyzed results were downloaded, and Spearman’s correlation heatmaps were visualized in R using the “pheatmap” package (v1.0.12). To avoid computational errors caused by a single algorithm and different sets of marker genes for TIL, we downloaded immune infiltrate data evaluated using the CIBERSORT (33), CIBERSORT-ABS (34), EPIC (35), MCP-counter (36), quantTIseq (37), xCell (38), TIMER (39), and TIDE (40) algorithms for the 33 cancer types from TCGA database using the TIMER2 web server (28). Similarly, immune infiltrate data evaluated using Tracking Tumor Immunophenotype (TIP) algorithms and immune activity score data for the 33 cancer types were downloaded from the TIP database. In addition, the single-sample gene set enrichment analysis (ssGSEA) based on the “gsva” package (v1.40.1) in R was also used to evaluate differences in the tumor-infiltrating fractions of 28 human immune cell phenotypes in the tumor microenvironment (41–43). Subsequently, Spearman’s correlations between FBXO22 expression and the level of TILs were calculated in R.

### 2.12 Statistical Analysis

Correlations between variables were explored using Spearman’s coefficients. Wilcoxon’s rank-sum tests were used to compare continuous variables between binary groups. Survival curves for categorical variable prognostic analyses were generated using the Kaplan-Meier method, while the log-rank test was used to estimate statistical significance. The significance level was set at *p* < 0.05, and all statistical tests were two-sided. All statistical data were analyzed using R or online analysis tools described in the relevant *Materials and Methods* subsections.

## 3 Results

### 3.1 Aberrant Expression of FBXO22 in Human Cancers is Associated With Poor Clinical Prognosis

First, we aimed to characterize the expression level of FBXO22 in different healthy tissues and cells, based on consensus data from the human protein atlas (HPA) database. The expression of FBXO22 gene was highest in the liver, followed by the placenta and adrenal gland, and lowest in the ductus deferens (**Supplementary Figure S1A**). Among the different cell types, cone and rod photoreceptor cells had the highest expression compared with other cell types, followed by early spermatids (**Supplementary Figure S1B**).

**Figure 1:**
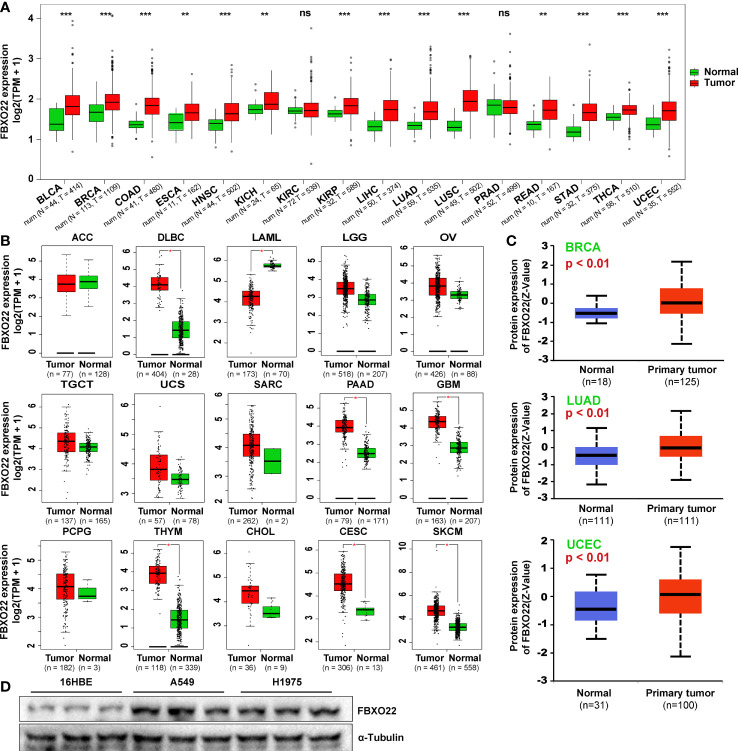
*FBXO22* expression in human tumors and healthy tissues. **(A)** *FBXO22* expression levels in BLCA, BRCA, COAD, ESCA, HNSC, KICH, KIRC, KIRP, LIHC, LUAD, LUSC, PRAD, READ, STAD, THCA, and UCEC from TCGA database. **(B)** Analysis of ACC, DLBC, LAML, LGG, OV, TGCT, UCS, SARC, PAAD, GBM, PCPG, THYM, CHOL, CESC, and SKCM in TCGA using GEPIA2; corresponding healthy tissues from the GTEx database were included as controls. **(C)** Total FBXO22 protein expression levels in healthy and primary breast cancer, lung adenocarcinoma, and UCEC tissue, based on the CPTAC dataset from UALCAN. **(D)** Total FBXO22 protein levels in human lung cancer and bronchial epithelial cell lines. ns, no significant difference; ^*^ *p* < 0.05; ^**^ *p* < 0.01; ^***^ *p* < 0.001.

To explore the role of FBXO22 expression in human cancer, we performed a pan-cancer analysis of FBXO22 using Gene Expression Quantification data of 33 cancers from TCGA database. Among the cancer types with a healthy sample number greater than ten, we observed high expression of FBXO22 in tissues from 14 tumor types (UCEC, ESCA, HNSC, KICH, READ, THCA, LIHC, KIRP, BLCA, BRCA, COAD, LUSC, STAD, and LUAD) compared with adjacent healthy tissues, but no significant difference in expression was observed in PRAD or KIRC (**Figure 1A**). For cancer types with no healthy samples or with a healthy sample number less than ten, we used the “Expression DIY” module of GEPIA2 web services to match GTEx datasets as controls. As shown in **Figure 1B**, the FBXO22 expression level in DLBC, PAAD, GBM, THYM, CESC, and SKCM was relatively high compared with that of corresponding healthy tissues; conversely, it was found to be low in LAML compared with corresponding healthy tissue. Furthermore, we noticed that the expression of FBXO22 also increased in LGG, OV, TGCT, USC, SARC, SARC, PCPG, and CHOL, though the difference was not statistically significant. To assess posttranslational levels of FBXO22, we analyzed FBXO22 protein expression using the CPTAC dataset in the UALCAN database. Consistent with our gene expression results, FBXO22 protein levels were highly expressed in BRCA, LUAD, and UCEC compared with healthy tissue (**Figure 1C**). FBXO22 was also upregulated in the NSCLC cell lines H1975 and A549 compared with the human bronchial epithelial cell line 16HBE (**Figure 1D**). Taken together, these results demonstrate that FBXO22 expression is upregulated in multiple cancers, implying that high FBXO22 expression levels may be associated with tumor progression.

**Figure 2:**
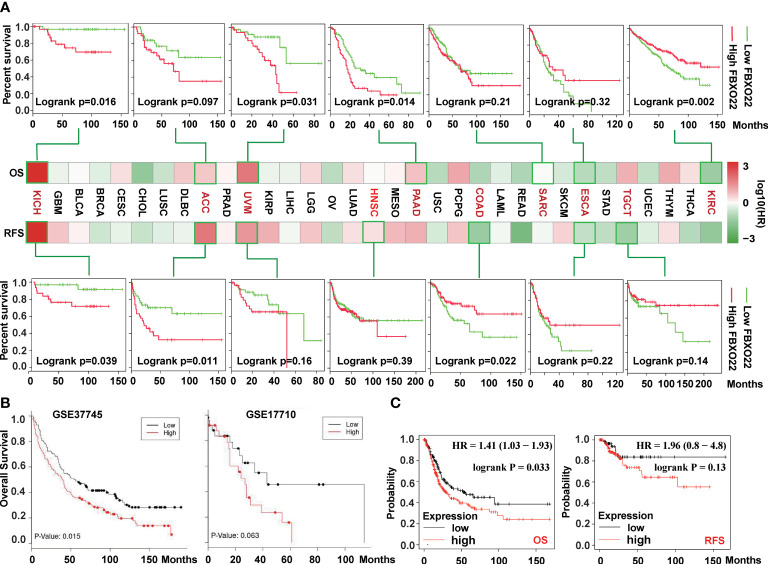
Correlation between survival and *FBXO22* gene expression in different cancer types. **(A)** Correlation between *FBXO22* gene expression and overall survival/disease-free survival in different tumor types in TCGA assessed using GEPIA2. **(B)** Correlation between *FBXO22* expression and overall survival in the lung cancer datasets GSE37745 and GSE17710 from the GEO database assessed using LUNG CANCER EXPLORER. **(C)** Correlation between *FBXO22* expression and overall and disease-free survival in pan-cancer using Kaplan-Meier analysis.

To explore the prognostic value of FBXO22 in people with different cancer types, we used the Survival Analysis module in GEPIA2 to analyze the correlation between FBXO22 expression and percent survival of people with different tumors. As shown in **Figure 2A**, high FBXO22 expression was associated with poor prognosis and lower OS in patients with KICH (Logrank *p* = 0.016), ACC (log-rank *p* = 0.097), UVM (log-rank *p* = 0.031), PAAD (log-rank *p* = 0.014), and SARC (log-rank *p* = 0.21). Conversely, low FBXO22 expression was associated with poor prognosis and OS in patients with ESCA (log-rank *p* = 0.32) or KIRC (log-rank *p* = 0.002). We also observed that high expression of the *FBXO22* gene was associated with poor RFS in KICH (log-rank *p* = 0.039), ACC (log-rank *p* = 0.011), UVM (log-rank *p* = 0.16), and HNSC (log-rank *p* = 0.39), but with better RFS in COAD (log-rank p =0.022), ESCA (log-rank *p* = 0.22), and TGCT (log-rank *p* = 0.14). Additionally, we used the LUNG CANCER EXPLORER service to perform tumor vs. normal and survival meta-analyses in different lung cancer datasets. We found that FBXO22 expression was positively associated with hazard rate (HR) in most datasets (**Supplementary Figures S2**–**S3**). High expression of FBXO22 was associated with poor OS in the lung cancer datasets GSE37745 (44) and GSE17710 (45) (**Figure 2B**). We then used Kaplan-Meier analysis tool to determine the relationship between pan-cancer and FBXO22 expression; as shown in **Figure 2C**, high expression of FBXO22 was linked to poor OS and RFS in pan-cancer. Taken together, these data indicate that FBXO22 expression is associated with poor prognosis in cancer.

### 3.2 FBXO22 Promotes Cell Cycle Progression

**Figure 3:**
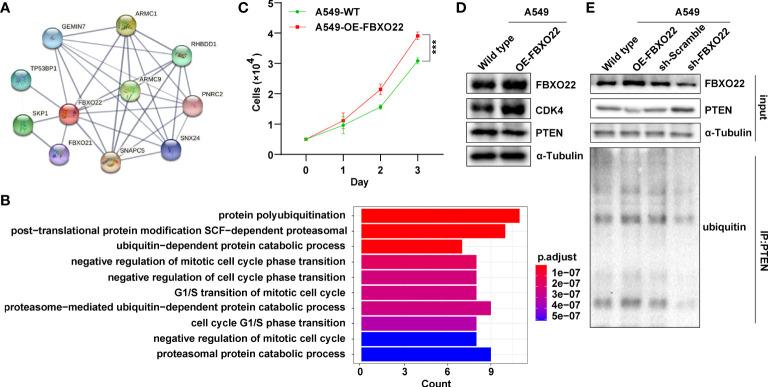
FBXO22 promotes cell cycle progression. **(A)** Network diagram of FBXO22-binding proteins obtained from the STRING database. **(B)** Based on the corresponding genes of the FBXO22-binding proteins, their associated biological processes were investigated using Gene Ontology (GO) enrichment analysis. **(C)** Growth of wild-type and *FBXO22*-overexpressing A549 cells. **(D)** *CDK4* expression in wild-type and *FBXO22*-overexpressing A549 cells. **(E)** Western blot analysis of PTEN protein levels and polyubiquitination in A549 cells stably expressing *FBXO22*, transfected with SH scramble or sh-FBXO22. ^***^ *p* < 0.001.

To explore the regulatory role of FBXO22 in tumorigenesis, we identified 10 FBXO22-binding proteins using the STRING database (**Figure 3A**). Next, we identified 13 proteins as substrates of FBXO22 ubiquitination and degradation (**Table 1**). FBXO22 regulates the levels of these proteins to alter tumor progression. Finally, we assessed the corresponding biological processes associated with these 23 FBXO22-associated proteins using GO enrichment analysis. We discovered that the genes encoding the FBXO2-associated proteins were significantly enriched in negative regulation of cell cycle and protein ubiquitination-related pathways (**Figure 3B**). To confirm this, a stable A549 cell line overexpressing FBXO22 was constructed. Overexpression of FBXO22 promoted proliferation in these cells (**Figure 3C**) and enhanced the level of cyclin-dependent kinase 4 (CDK4) (**Figure 3D**). This indicates that FBXO22 is related to the cell cycle regulation. Notably, we observed decreased levels of PTEN, a FBXO22 substrate, in FBXO22-overexpressing cells (**Figure 3D**). Subsequently, we explored the potential regulation of PTEN by FBXO22 *via* ubiquitination. We immunoprecipitated PTEN and found that the level of ubiquitinated PTEN was increased in FBXO22-overexpressing cells compared with wild type (**Figure 3E**). Conversely, the ubiquitination level of PTEN was decreased after interfering FBXO22 (**Figure 3E**). This demonstrates that FBXO22 affects the progression of NSCLC by regulating PTEN ubiquitination levels.

**Table 1:** Target substrates for FBXO22 degradation.

Substrate	Description	Reference
KDM4A	Lysine demethylase 4A	(3)
KDM4B	Lysine demethylase 4B	(5)
p53	Tumor protein P53	(4)
P21 (CDKN1A)	Cyclin-dependent kinase inhibitor 1A	(1)
KLF4	Kruppel-like factor 4	(8)
LKB1 (STK11)	Liver kinase B1	(10)
BSG	Basigin (Ok blood group)	(9)
BACH1	BTB domain and CNC homolog 1	(11)
PTEN	Phosphatase and tensin homolog	(12)
SNAI1	Snail family transcriptional repressor 1	(7)
HDM2	MDM2 proto-oncogene	(6)
BAG3	BAG cochaperone 3	(13)
PHLPP1	PH domain and leucine-rich repeat protein phosphatase 1	(14)

### 3.3 Genetic Alteration of FBXO22 in Human Cancers Is Associated With Good Clinical Prognosis

**Figure 4:**
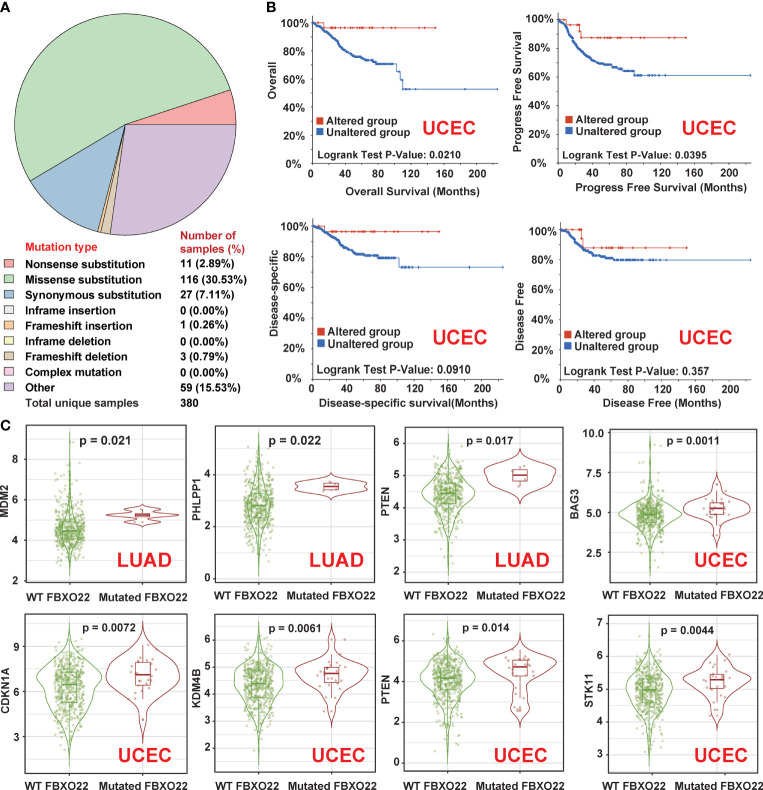
Genetic alterations in *FBXO22* in different cancer types. **(A)** The distribution of different types of mutation in *FBXO22* displayed using the COSMIC tool. **(B)** Potential correlation between *FBXO22* alteration status and overall, progression-free, disease-free, and disease-specific survival in UCEC, as analyzed using the cBioPortal tool. **(C)** *MDM2*, *PHLPP1*, and *PTEN* expression in samples with wild-type or mutated *FBXO22* in LUAD; *CDKN1A*, *KDM4B*, *STK11*, and *PTEN* expression in samples with wild-type or mutated *FBXO22* in UCEC.

Genetic alterations in FBXO22 may affect its function. Hence, we explored the genetic alteration status of FBXO22 in human cancers. First, we explored the distribution of different mutation types for FBXO22 using COSMIC. As shown in **Figure 4A**, a missense substitution is the most commonly observed mutation type, observed in the 30.53% of the cancer types. We also used the cBioPortal tool to analyze the genetic alteration status of FBXO22 in different cancer types from the TCGA cohorts. We observed the highest FBXO22 alteration frequency among patients with UCEC, followed by mesothelioma (**Supplementary Figure S4A**). Notably, the FBXO22 alterations in mesothelioma were all identified as “Amplification” (**Supplementary Figure S4A**). Additionally, we found that the frequency of arginine to histidine or cysteine mutations at position 96 in the FBXO22 protein was the highest among all mutations (**Supplementary Figure S4B**). Next, we examined the association between genetic alterations in FBXO22 and clinical outcomes. We discovered that altered FBXO22 was associated with better prognosis in terms of OS (*p* = 0.021), progression-free (*p* = 0.0395), and disease-specific (*p* = 0.091) survival, but not disease-free (*p* = 0.357) survival, compared with cases without FBXO22 alteration among UCEC cases (**Figure 4B**). This implies that alterations in FBXO22 may be beneficial for UCEC patients.

Finally, we utilized the “gene_Mutation” module in TIMER2 to compare the substrate gene expression according to FBXO22 mutation status. We found that FBXO22 mutation was correlated with the expression of at least one substrate gene in COAD, GBM, LUAD, LUSC, SKCM, STAD, and UCEC (**Figure 4C** and **Supplementary Figures S4C, D**). We noted an increased expression of MDM2, PHLPP1, and PTEN in LUAD samples with mutated FBXO22 and an increased expression of CDKN1A, KDM4B, STK11, and PTEN in UCEC samples with mutated FBXO22 (**Figure 4C**). These findings suggest that mutant FBXO22 may lose E3 ligase activity, especially in people with UCEC and LUAD. This also explains why FBXO22 mutation was associated with better prognosis in patients with UCEC.

### 3.4 Low Methylation of FBXO22 Is Related to Poor Prognosis

**Figure 5:**
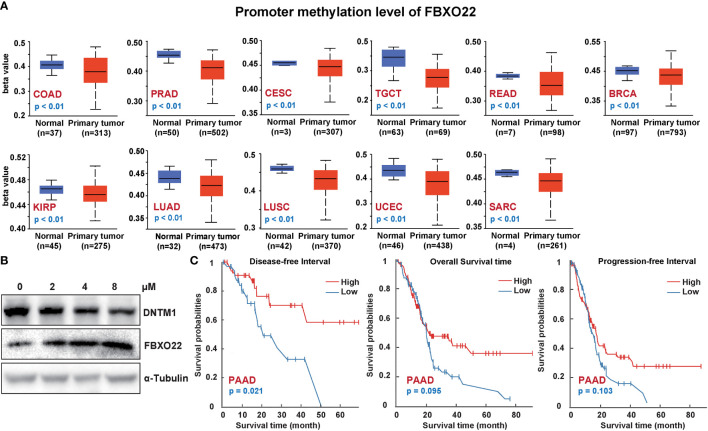
*FBXO22* DNA methylation in different cancer types. **(A)** *FBXO22* methylation levels in COAD, PRAD, CESC, TGCT, READ, KIRP, LUAD, LUSC, BRCA, UCEC, and SARC. **(B)** FBXO22 expression was decreased in A549 cells treated with different concentrations of SGI-1027, a DNA methyltransferase inhibitor. **(C)** Potential correlation between *FBXO22* methylation and overall, progression-free, and disease-specific survival.

Next, we explored whether FBXO22 methylation was associated with clinical prognosis. We used the UALCAN tool to analyze FBXO22 methylation in various cancers from TCGA database. We found that the level of methylation in the FBXO22 promoter region was significantly decreased in COAD, PRAD, CESC, TGCT, READ, KIRP, LUAD, LUSC, BRCA, UCEC, and SARC compared with normal tissues (**Figure 5A**). This may be related to the high FBXO22 expression in multiple cancer types. Indeed, inhibiting the expression of DNA methyltransferase 1 (DNTM1) in A549 cells resulted in elevated FBXO22 (**Figure 5B**). We subsequently explored whether FBXO22 methylation was associated with prognosis among people with cancer using the DNMIVD tool. We found that hypermethylation of FBXO22 was related to poor OS, disease-free interval, and progression-free interval in PAAD (**Figure 5C**). Similarly, the association of methylated CpG islands and prognosis was analyzed using DNMIVD. Feature importance score calculation using the xgboost algorithm revealed 3 significant methylated CpGs: cg00942495, cg05374463, and cg08290738 (**Supplementary Figure S5A**). We identified a correlation between low cg08290738 methylation and poor OS in CESC and ESCA (**Supplementary Figure S5B**; high levels of cg00942495 or cg05374463 methylation were also found to be associated with better survival in THYM (**Supplementary Figure S5D**).

### 3.5 FBXO22 Expression Associates With the Tumor Immune Infiltrate

TILs are an important part of tumor microenvironment (TME) and are involved in the occurrence, development, and metastasis of tumors (46). However, the relationship between FBXO22 expression and TILs/TME is unclear. To understand the relationship between FBXO22 and the immune composition of tumors, we first used the TISIDB to evaluate the relationship between TIL abundance and FBXO22 expression, CNV, and methylation. As shown in **Supplementary Figure S6**, the abundance of most TIL types was positively correlated with FBXO22 expression in both GBM and UVM cancers; however, the abundance of almost all TIL types in other cancers was negatively correlated with expression of FBXO22 (**Supplementary Figure S6A**). There was a weak correlation between changes in FBXO22 CNV and TIL levels (**Supplementary Figure S6B**). The methylation of FBXO22 was strongly positively correlated with the abundance of TILs in PRAD and TGCT (**Supplementary Figure S6C**). These lines of evidence suggest that the expression level and genetic alterations of FBXO22 in the tumor may influence antitumor immunity. The activity of the tumor immune cycle is a direct integrated manifestation of the functions of the chemokine system and other immune regulators (31, 32). Thus, we used the TISIDB to analyze correlations between the FBXO22 expression and chemokines and their receptors across human cancers. We found a significant negative correlation between FBXO22 expression and chemokines or receptors in almost all cancers (**Supplementary Figures S7A, B**). Similar analyses have found that three kinds of immunomodulators (immunoinhibitors, immunostimulators, and MHCs) were also significantly inversely correlated with FBXO22 expression (**Supplementary Figures S7C–E**). Taken together, this evidence suggests that tumor FBXO22 expression plays an important role in tumor immune regulation.

**Figure 6:**
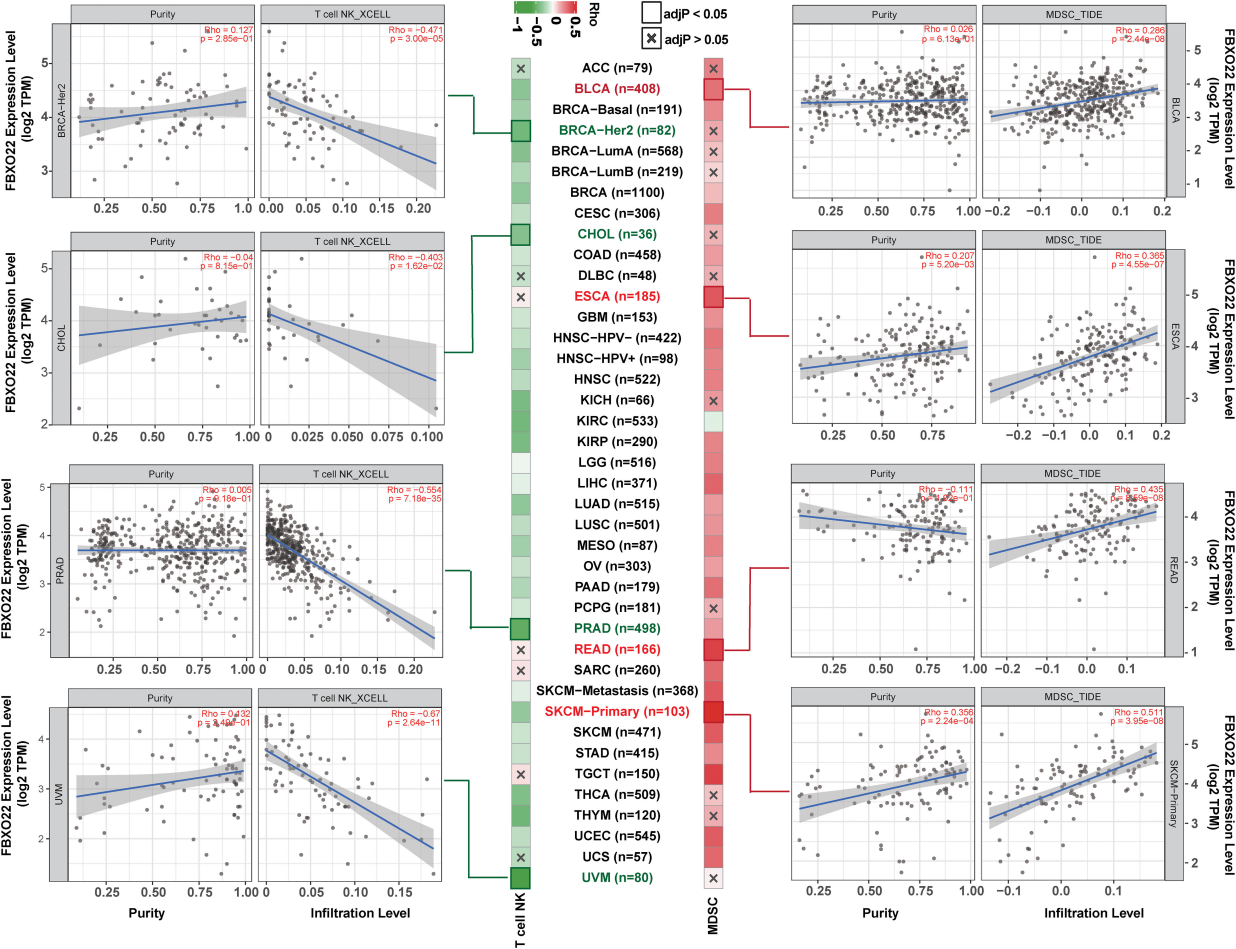
Natural killer (NK) T-cell and myeloid-derived suppressor cells (MDSCs) in different tumor tissues from TCGA. The correlation between *FBXO22* expression and NK T-cell infiltration levels was estimated using XCELL algorithms, and the MDSC infiltration levels were estimated using TIDE algorithms.

To avoid computational errors arising from the use of a single algorithm and different marker gene sets of TIICs, we utilized the CIBERSORT, CIBERSORT-ABS, EPIC, MCP-counter, quanTIseq, TIMER, TIP, and xCell algorithms to evaluate the correlation between FBXO22 expression and the level of immune infiltration of TILs. Although there were differences in the levels of immune cell infiltration calculated using different algorithms, we noted that the calculations produced by at least five of the algorithms showed that B-cell and macrophage infiltration levels were negatively correlated with FBXO22 expression in multiple cancers, including LUAD, LUSC, and SARC (**Supplementary Figures S8A–H**). We discovered significant negative correlations between FBXO22 expression and the NK T-cell infiltration level as estimated by the XCELL algorithm in many types of cancer, including BRCA-Her2 (Rho = −0.471), CHOL (Rho = −0.403), PRAD (Rho = −0.554), and UVM (Rho = −0.67) (**Figure 6**). Similarly, we found a significant positive correlation between FBXO22 expression and the MDSC infiltration level, as estimated by TIDE algorithm, including in BLCA (Rho = 0.286), ESCA (Rho = 0.365), READ (Rho = 0.435), and SKCM-primary (Rho = 0.511) (**Figure 6**). NK T cells enhance the function of dendritic cells (DCs), T cells, B cells, and other immune cells by secreting various cytokines and chemokines (47). MDSCs may not only inhibit the antitumor immune response but also directly stimulate tumor growth and metastasis (48). These results suggest that high FBXO22 expression in tumors may be detrimental to the antitumor immune response.

**Figure 7:**
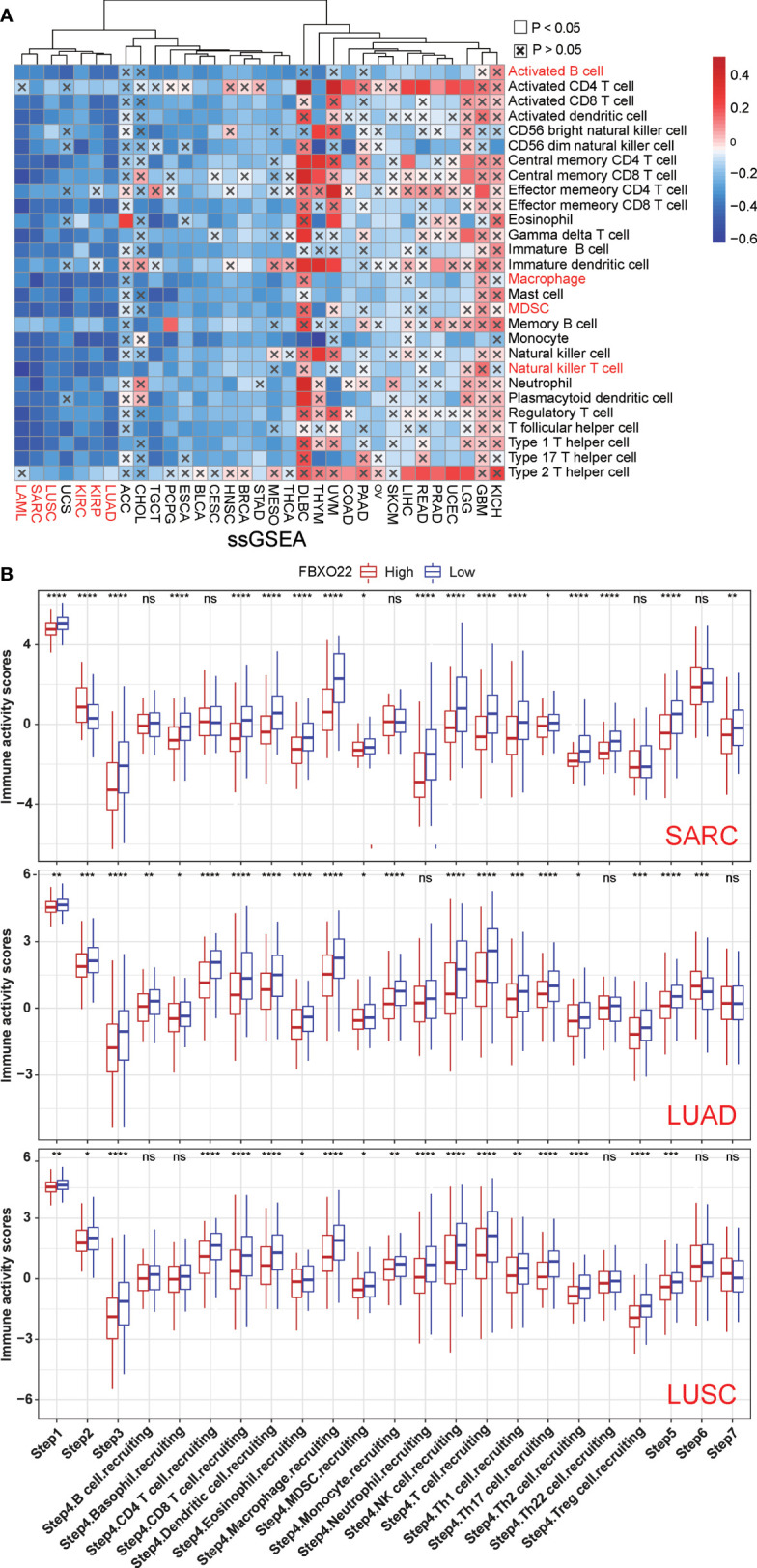
The effect of FBXO22 on immunological status in pan-cancer. **(A)** Correlation between FBXO22 and 28 tumor-associated immune cells, as calculated using the ssGSEA algorithm. **(B)** Differences in the various steps of the cancer immune cycle between groups with high and low *FBXO22* expression. ns, no significant difference; ^*^ *p* < 0.05; ^**^ *p* < 0.01; ^***^ *p* < 0.001; ^****^ *p* < 0.0001.

Antitumor immune responses can be conceptualized as a series of stepwise events including the release of cancer cell antigens (step 1), cancer antigen presentation (step 2), priming and activation (step 3), trafficking of immune cells to tumors (step 4), infiltration of immune cells into tumors (step 5), recognition of cancer cells by T cells (step 6), and killing of cancer cells (Step 7) (32). Xu and colleagues evaluated the activities in these steps using ssGSEA based on gene expression in individual samples (32). Therefore, we performed ssGSEA to decipher the involvement of FBXO22 in the immune activation process. We found that FBXO22 expression in LUAD, LUSC, KIRP, KIRC, SARC, and LAML were negatively correlated with the levels of infiltration of multiple immune cells (**Figure 7A**). Notably, these immune response activation steps were significantly activated when FBXO22 expression was low in LUAD, LUSC, and SARC (**Figure 7B**). Together, these data suggest that FBXO22 expression in these tumors may play a critical role in the immune response to tumors.

## 4 Discussion

Aberrant posttranslational modification (PTM) can lead to inappropriate regulation of protein levels and promote tumorigenesis. Degradation of proteins by ubiquitylation is an important mechanism of PTM and is involved in multiple diseases, including multiple types of cancer. Ubiquitinated proteins are degraded *via* the proteasome, and this alters their localization, affects their activity, and promotes or interferes with protein interactions, thereby affecting the regulation of cellular events such as proliferation, apoptotic death, and cell cycle progression (49). Dysregulation of protein ubiquitination is a cause of aberrant changes in tumor suppressor or oncogene expression. FBXO22 has been identified to be critically involved in regulating the ubiquitination of substrate proteins, thus regulating tumor progression. However, until now, it has been unclear whether FBXO22 plays a critical role in the pathogenesis of different cancers *via* a common molecular mechanism. In this study, we explored the correlations between clinical prognosis and FBXO22 expression, methylation, and mutation in 33 cancer types from the TCGA database using bioinformatic analysis.

The results of our analysis showed that FBXO22 was highly expressed in multiple cancer types. Nevertheless, the impact of FBXO22 expression on survival outcomes varied depending on the cancer type, and variation in data sources may also lead to variable results for the same cancer type. For example, we used the LUNG CANCER EXPLORER tool to perform survival analysis in the lung cancer datasets GSE37745 (44) and GSE17710 (45) in which we observed a significant inverse correlation between FBXO22 expression and OS. However, when we used the GEPIA2 tool to perform survival analysis in the lung cancer datasets LUAD and LUSC from the TCGA, we discovered no such correlations between FBXO22 expression and OS. Such conflicting results may be due to differences in data processing and updates in survival data between the datasets. However, in a pan-cancer context, we found that high FBXO22 expression was associated with poor OS and RFS, as determined using Kaplan-Meier analysis. Therefore, overall, we can conclude that high FBXO22 expression resulted in worse survival outcomes in most cancer types.

Although many studies have shown that FBXO22 is involved in oncogenesis owing to its ubiquitination activity and the subsequent degradation of multiple proteins (2), the means by which FBXO22 affects tumor growth by regulating substrate protein levels was largely unknown. Thus, we identified 23 FBXO22-binding proteins using the STRING database and literature search; these were all significantly enriched in cell cycle-negative regulation and ubiquitination pathways. Therefore, we propose that FBXO22 controls the cell cycle by regulating substrate protein levels, which we confirmed in the lung cancer cell line A549: overexpression of FBXO22 increased the expression of cyclin-dependent kinase 4 (CDK4) and promoted cell proliferation. We also found that FBXO22 regulates the level of the substrate PTEN by ubiquitination in A549 cells. However, previous studies have shown that FBXO22 does not only promote cell proliferation but also inhibit migration and metastasis in lung and breast cancer (6, 7, 11), indicating that FBXO22 has both oncogenic and tumor suppressive roles. This may depend on the function and levels of the substrate.

The genetic alteration of FBXO22 may affect the expression level of substrates. The highest alteration frequency of FBXO22, in which “mutation” was the primary type, appears in patients with UCEC, as established using the cBioPortal tool; we discovered that those with this alteration in FBXO22 had a better prognosis in terms of overall, progression-free, and disease-specific survival compared with those without the FBXO22 alteration. Notably, the expression of FBXO22 in UCEC patients was not significantly associated with OS or RFS. This may be because, in a subset of cancer types, changes in FBXO22 expression levels alone are not sufficient to affect tumor progression, and genetic alterations in FBXO22 have a more pronounced effect on tumors. Indeed, mutations in FBXO22 in UCEC were accompanied by increased expression of multiple substrate genes in our analysis, including CDKN1A, KDM4B, STK11, and PTEN. Therefore, genetic alterations in FBXO22 have a greater impact on UCEC specifically.

DNA methylation is one of the most abundant and well-studied epigenetic modifications, playing an essential role in tumorigenesis (50). Using the UALCAN tool, we observed that the FBXO22 promoter methylation level was significantly decreased in multiple cancer tissues compared with healthy tissues. This may be an important explanation for the high level of FBXO22 expression in a variety of cancer types. Unfortunately, we did not retrieve literature reports on FBXO22 methylation. In our study, we confirmed that suppression of DNTM1 in A549 cells increased the expression level of FBXO22. This implies a potential link between FBXO22 expression and methylation.

The number, localization, and phenotypes of TILs have an important effect on cancer progression (51). We found that FBXO22 expression was negatively correlated with the abundance of multiple TIL types, including NK T cells, in most cancer types, whereas FBXO22 methylation was positively correlated with the abundance of TILs, as established using TISIDB database analysis tools. We also confirmed that high expression of FBXO22 was associated with lower TIL levels in some cancer types, including LUAD, LUSC, and SARC, as established using different evaluation algorithms. Considering the complexity of the processes involved in the tumor immune response, we evaluated the immune activation steps using the TIP algorithm across 33 cancer types, and determined that individuals with low FBXO22 expression levels had higher activation scores, especially in LUAD, LUSC, and SARC. Notably, our findings are the first to indicate a correlation between FBXO22 expression and immune infiltration. In the future, it will be important to investigate the specific role of FBXO22 in cancer immune regulation, especially in LUAD, LUSC, and SARC.

## 5 Conclusions

Taken together, our pan-cancer analyses revealed correlations between FBXO22 expression, methylation, mutation and clinical prognosis, and immune cell infiltration, which contribute to a better understanding of the role of FBXO22 in tumorigenesis. In future studies, the potential relationship between FBXO22 methylation and expression and antitumor immunity warrants in-depth exploration. Our analysis provided a relatively comprehensive understanding of the oncogenic role of FBXO22 in different tumor types. In addition, FBXO22 has been discussed as a new potential therapeutic target for hepatocellular carcinoma (1); based on our analysis, FBXO22 may be a valuable drug target for multiple cancer therapies.

## Data Availability Statement

The original contributions presented in the study are included in the article/**Supplementary Material**. Further inquiries can be directed to the corresponding authors.

## Author Contributions

YW and XC designed this work. SC and SM analyzed the data. HW, JY, BD, ZG, and YM helped or performed experiments and analyses. XC helped in the revision of the manuscript. SC and SM wrote the manuscript.

## Funding

This work was supported by grants from the National Science and Technology Major Project of China (Grant No. 2019ZX09201004-001).

## Conflict of Interest

The authors declare that the research was conducted in the absence of any commercial or financial relationships that could be construed as a potential conflict of interest.

## Publisher’s Note

All claims expressed in this article are solely those of the authors and do not necessarily represent those of their affiliated organizations, or those of the publisher, the editors and the reviewers. Any product that may be evaluated in this article, or claim that may be made by its manufacturer, is not guaranteed or endorsed by the publisher.

## Supplementary Material

Supplementary Figure 1
*FBXO22* expression levels in healthy tissues and cells. **(A)** Consensus Normalized eXpression (NX) levels of *FBXO22* in 55 tissue types and six blood cell types, created by combining the data from the three transcriptomics datasets (HPA, GTEx and FANTOM5) using the internal normalization pipeline. **(B)** NX *FBXO22* levels in different cell types.

Supplementary Figure 2 Graph showing tumor versus normal meta-analysis of data with the *FBXO22* gene and tumor type using LUNG CANCER EXPLORER.

Supplementary Figure 3 Graph showing survival meta-analysis of data with the *FBXO22* gene and tumor type using LUNG CANCER EXPLORER.

Supplementary Figure 4 Features of *FBXO22* genetic alterations in different human cancer types from the TCGA, as displayed using the cBioPortal tool. **(A)** The frequency of different *FBXO22* genetic alterations in different tumor types in TCGA. **(B)** Statistics associated with FBXO22 mutation sites in different tumor types. **(C)** Heatmap of differentially expressed substrate genes between the mutated and wild-type *FBXO22* in different cancer types. **(D)** *BACH1, CDKN1A* and *MDM2* expression in samples with wild-type or mutated *FBXO22* in GBM; *BAG3, TP53* and *BACH1* expression in samples with wild-type or mutated *FBXO22* in COAD; *CDKN1A* expression in samples with wild-type or mutated *FBXO22* in LUSC and SKCM.

Supplementary Figure 5 Spearman correlation heatmap showing the relationship between survival prognosis and methylated CpG islands in *FBXO22*. **(A)** The feature importance score for methylated CpGs in *FBXO22* was calculated using the xgboost algorithm in cancers from TCGA. **(B)** Correlation between overall survival and methylated cg08290738 in CESC and ESCA. **(C, D)** Correlation between overall survival and methylated cg00942495 and cg05374463 sites in THYM.

Supplementary Figure 6 Spearman correlation heatmap showing the relationship between abundance of tumor-infiltrating lymphocytes (TILs) and *FBXO22* expression, copy number variation (CNV), and methylation in TISIDB database. Spearman correlations between **(A)** *FBXO22* expression, **(B)** *FBXO22* CNV, and **(C)** *FBXO22* methylation and TIL abundance.

Supplementary Figure 7 Spearman correlation heatmap showing the relationship between *FBXO22* expression and, three kinds of immunomodulators, and chemokines. Spearman correlations between expression of *FBXO22* and **(A)** chemokines, **(B)** receptors, **(C)** immunoinhibitors, **(D)** immunostimulators, and **(E)** major histocompatibility complex components across human cancers.

Supplementary Figure 8 Spearman correlation heatmap showing the relationship between *FBXO22* expression and tumor infiltrating leukocytes (TILs) based on different algorithms. **(A)** CIBERSORT-ABS, **(B)** CIBERSORT, **(C)** EPIC, **(D)** TIMER, **(E)** MCP-counter, **(F)** quantTIseq, **(G)** xCell, and **(H)** TIP.
