# Supplementary figures and images for: Pan-Cancer Analyses Reveal Oncogenic Role and Prognostic Value of F-Box Only Protein 22
Source: Front Oncol. 2022 Jan 24;11:790912. doi: 10.3389/fonc.2021.790912 (PMC8818750; doi:10.3389/fonc.2021.790912)

**A****Consensus dataset**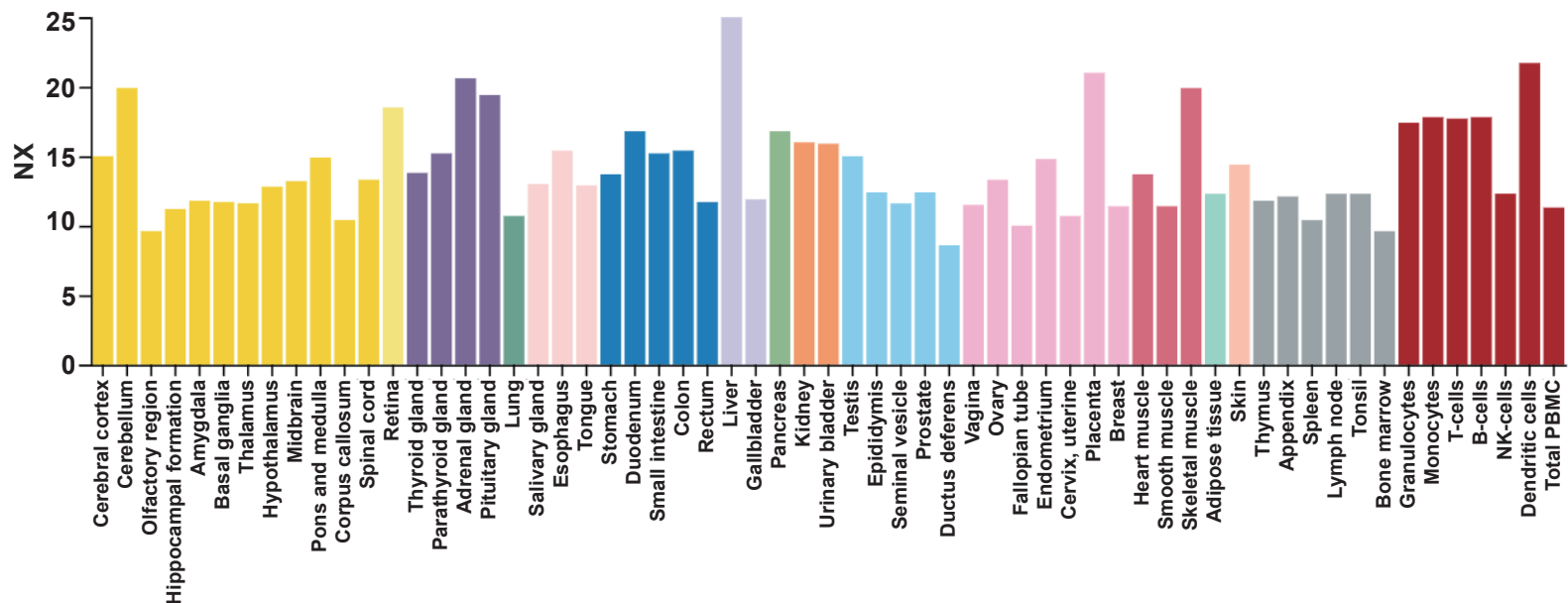**B****Single cell types**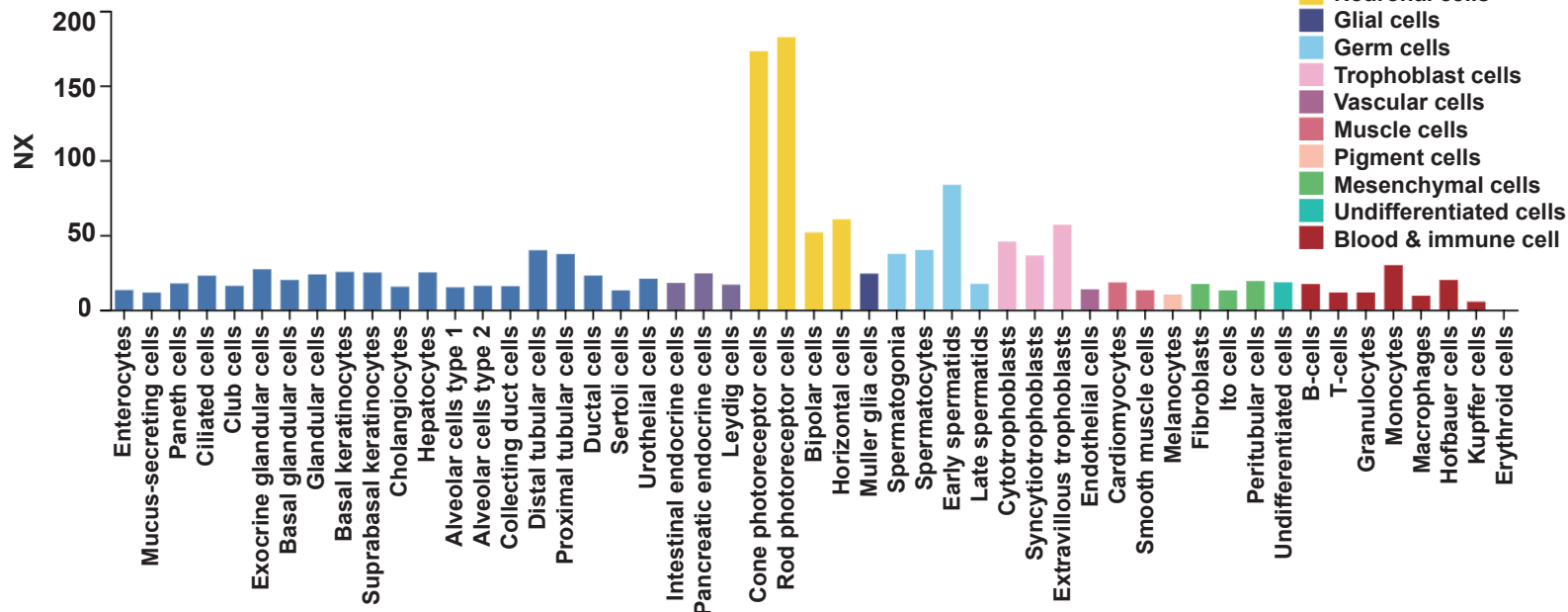

Supplement: Supplementary Figure 1 — FBXO22 expression levels in healthy tissues and cells. (A) Consensus Normalized eXpression (NX) levels of FBXO22 in 55 tissue types and six blood cell types, created by combining the data from the three transcriptomics datasets (HPA, GTEx and FANTOM5) using the internal normalization pipeline. (B) NX FBXO22 levels in different cell types. [file Image_1.pdf]

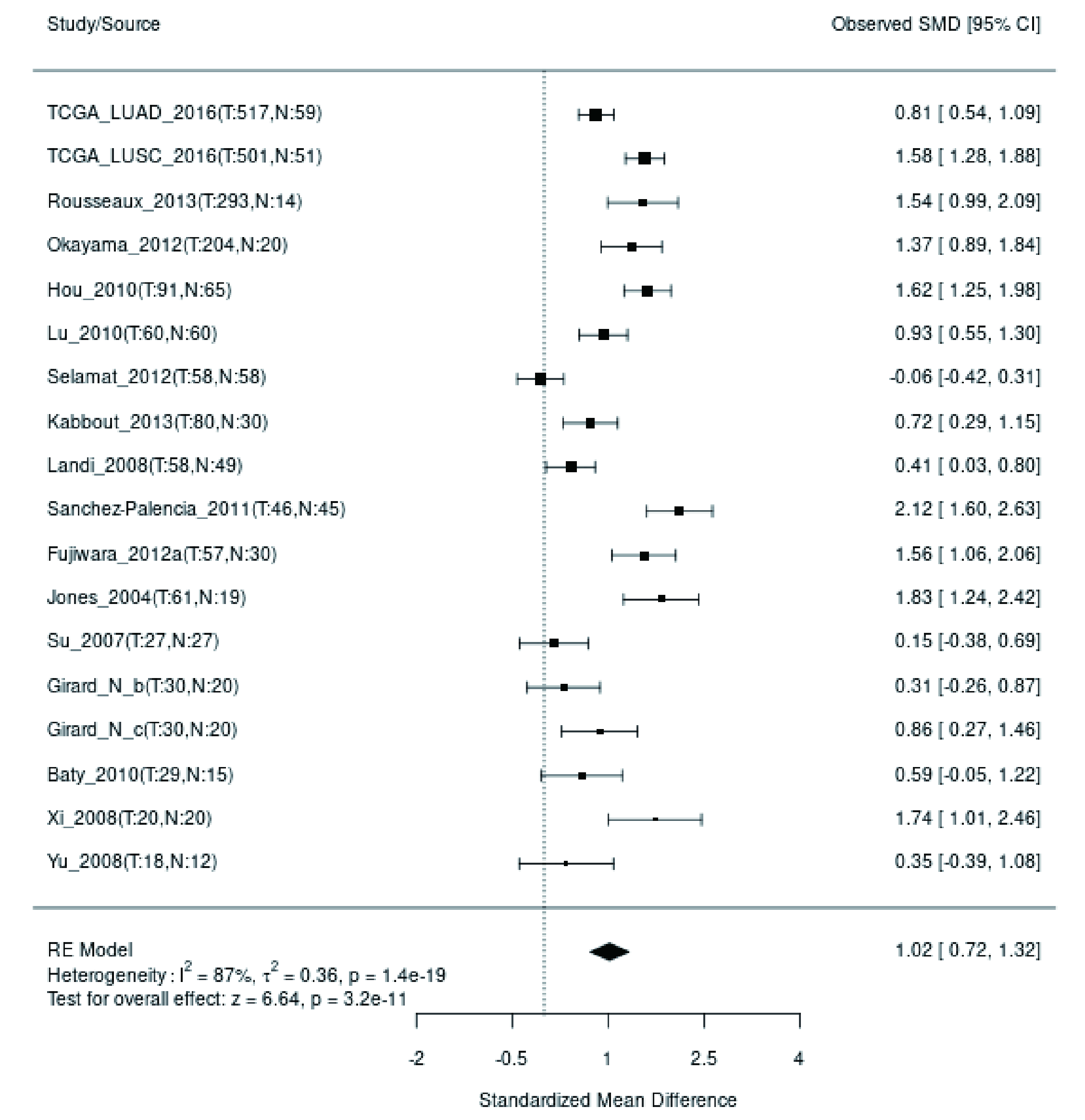

Supplement: Supplementary Figure 2 — Graph showing tumor versus normal meta-analysis of data with the FBXO22 gene and tumor type using LUNG CANCER EXPLORER. [file Image_2.tif]

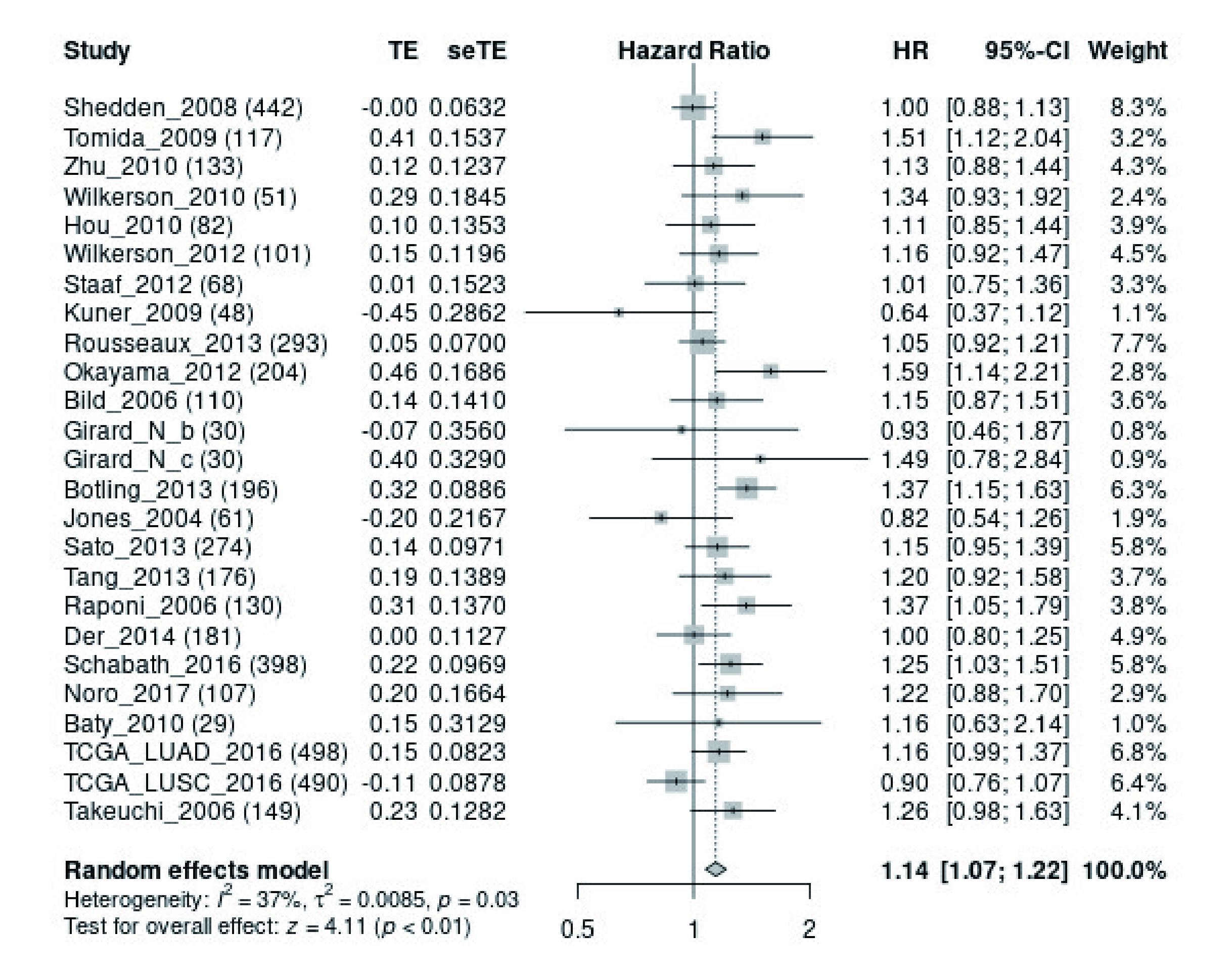

Supplement: Supplementary Figure 3 — Graph showing survival meta-analysis of data with the FBXO22 gene and tumor type using LUNG CANCER EXPLORER. [file Image_3.tif]

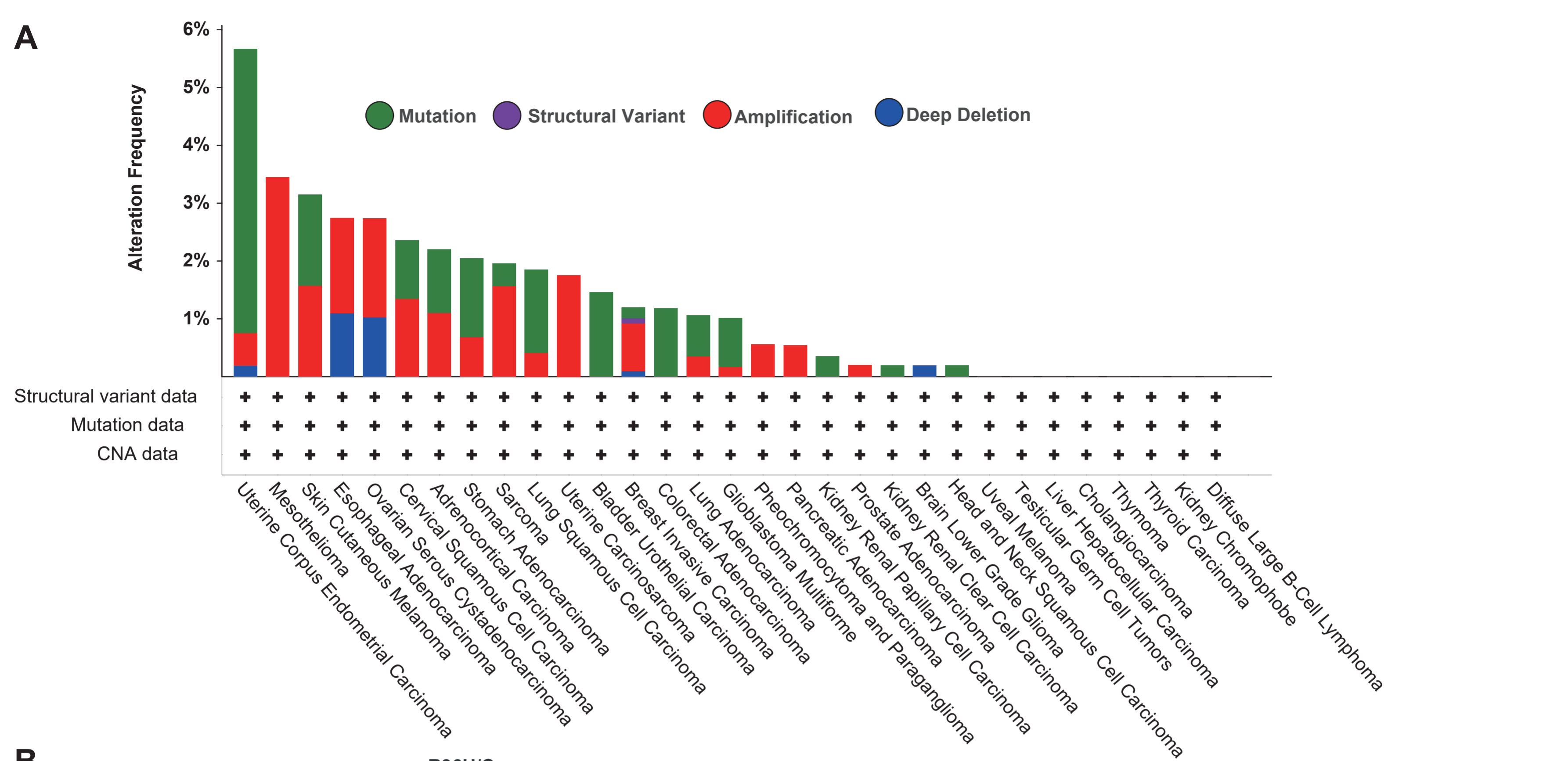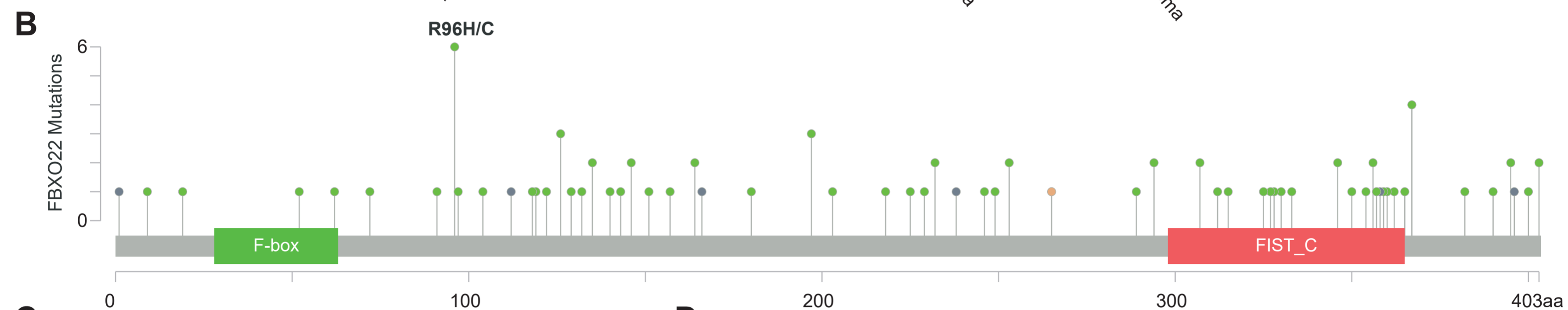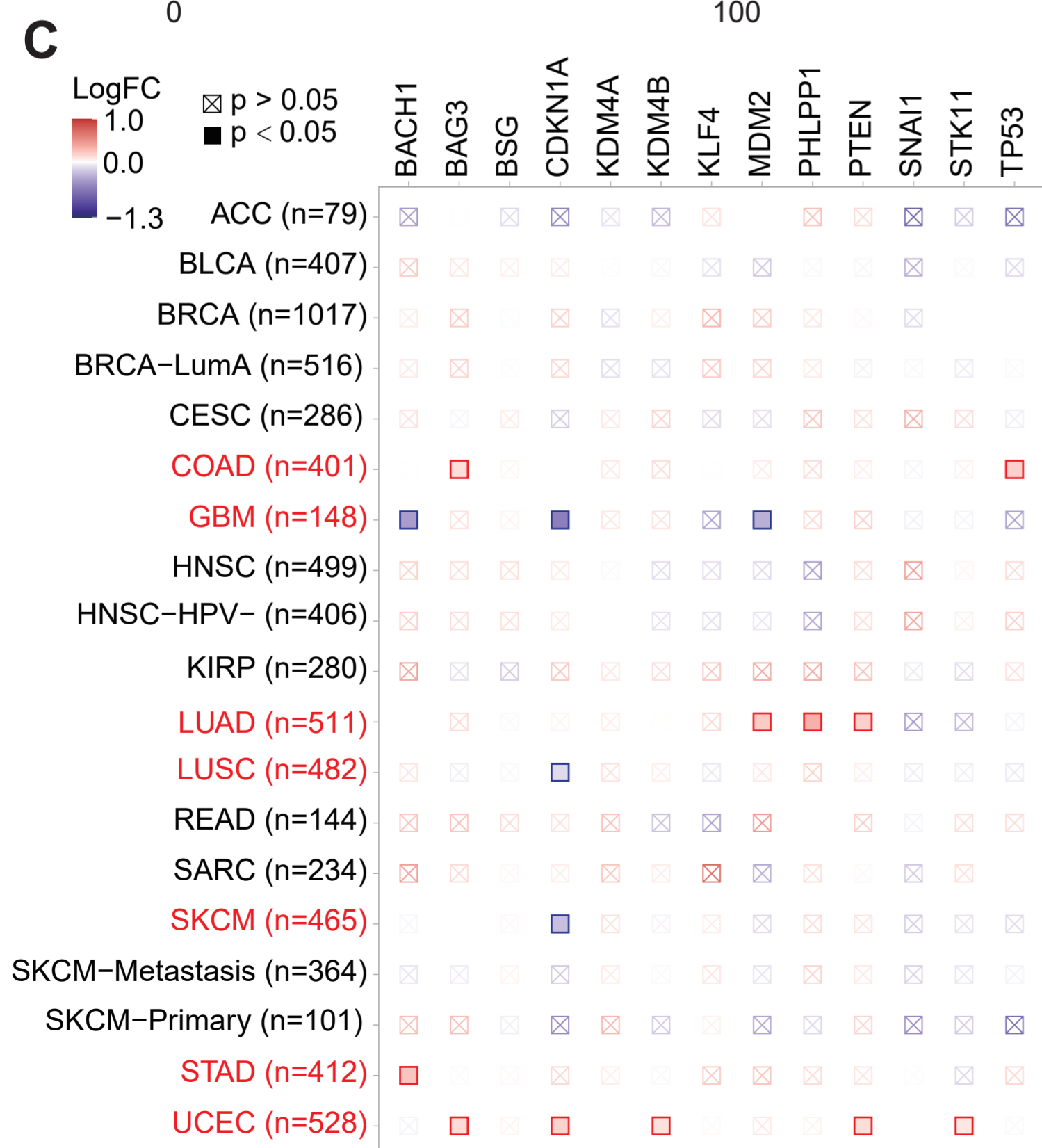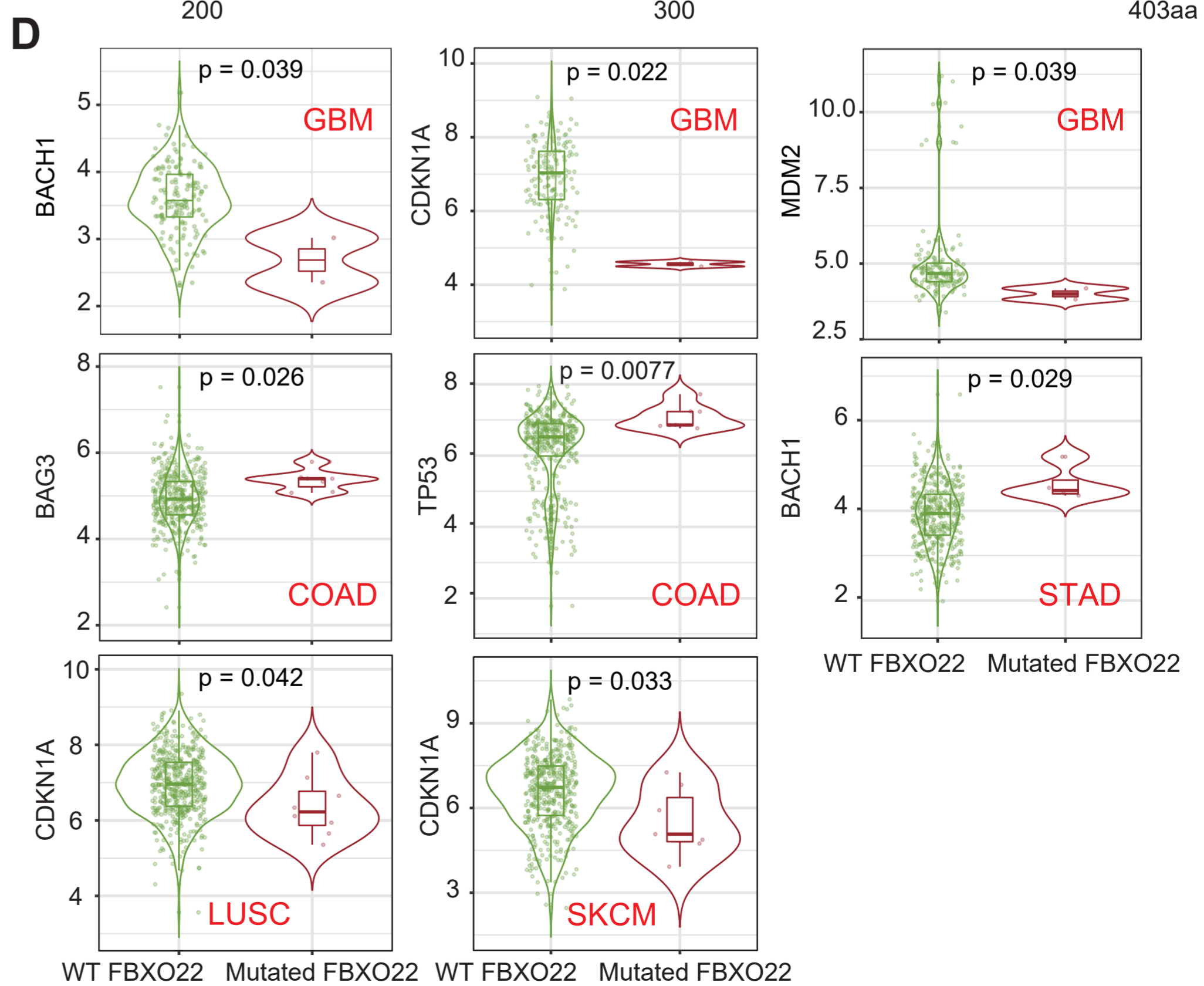

Supplement: Supplementary Figure 4 — Features of FBXO22 genetic alterations in different human cancer types from the TCGA, as displayed using the cBioPortal tool. (A) The frequency of different FBXO22 genetic alterations in different tumor types in TCGA. (B) Statistics associated with FBXO22 mutation sites in different tumor types. (C) Heatmap of differentially expressed substrate genes between the mutated and wild-type FBXO22 in different cancer types. (D) BACH1, CDKN1A and MDM2 expression in samples with wild-type or mutated FBXO22 in GBM; BAG3, TP53 and BACH1 expression in samples with wild-type or mutated FBXO22 in COAD; CDKN1A expression in samples with wild-type or mutated FBXO22 in LUSC and SKCM. [file Image_4.pdf]

**A**

Ranking of importance

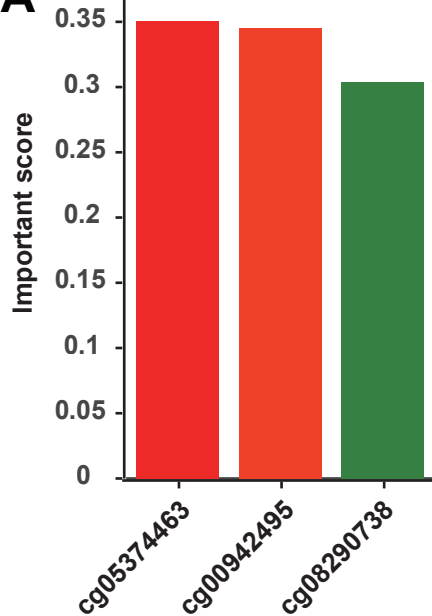**B**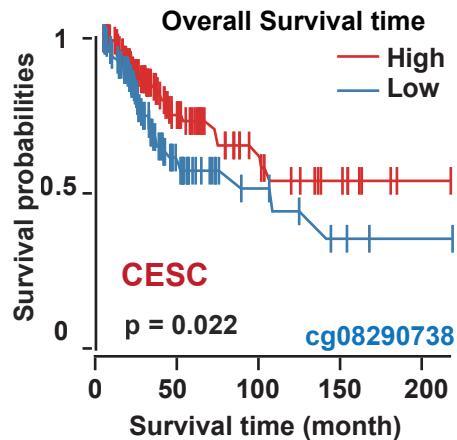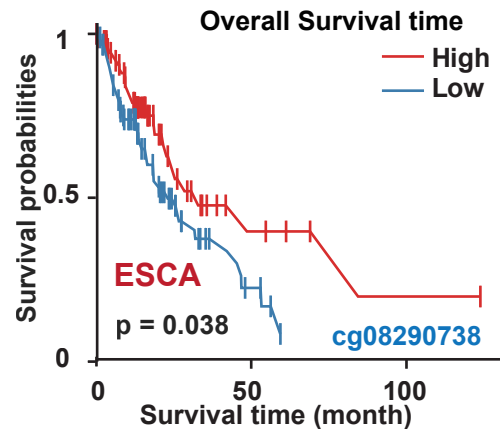**C**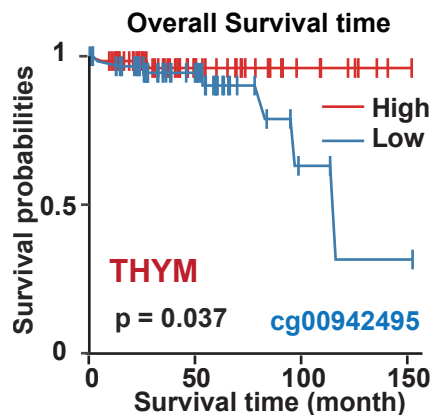**D**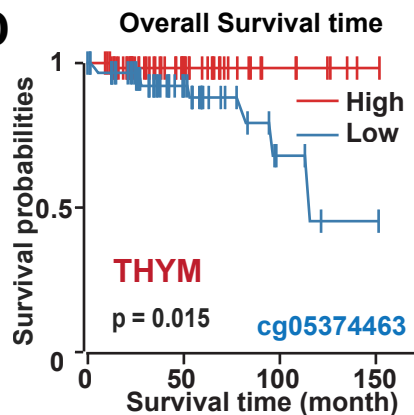

Supplement: Supplementary Figure 5 — Spearman correlation heatmap showing the relationship between survival prognosis and methylated CpG islands in FBXO22. (A) The feature importance score for methylated CpGs in FBXO22 was calculated using the xgboost algorithm in cancers from TCGA. (B) Correlation between overall survival and methylated cg08290738 in CESC and ESCA. (C, D) Correlation between overall survival and methylated cg00942495 and cg05374463 sites in THYM. [file Image_5.pdf]

**A**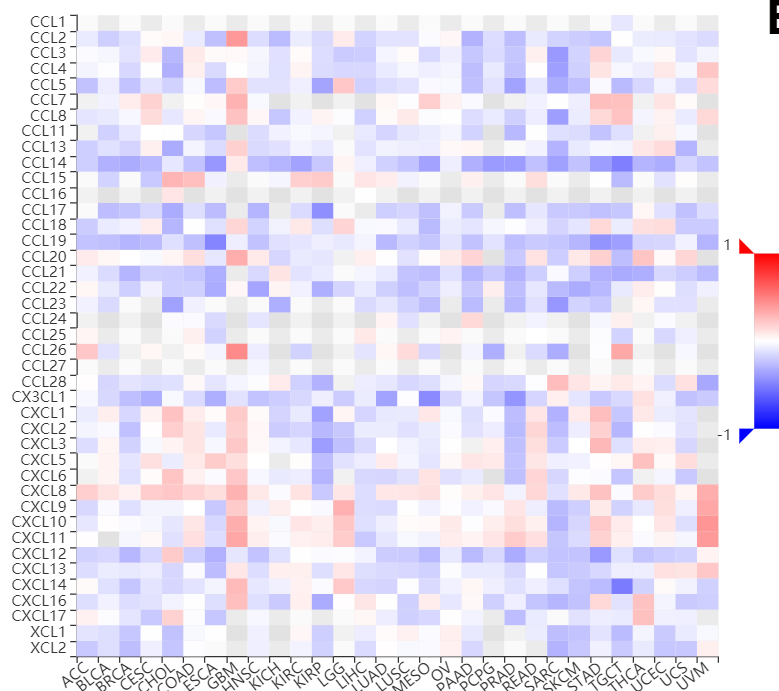**B**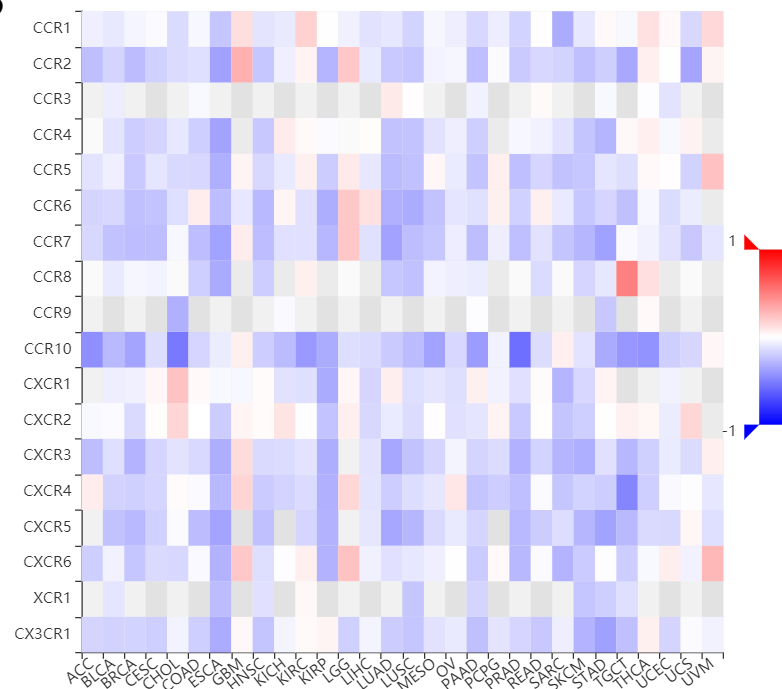**C**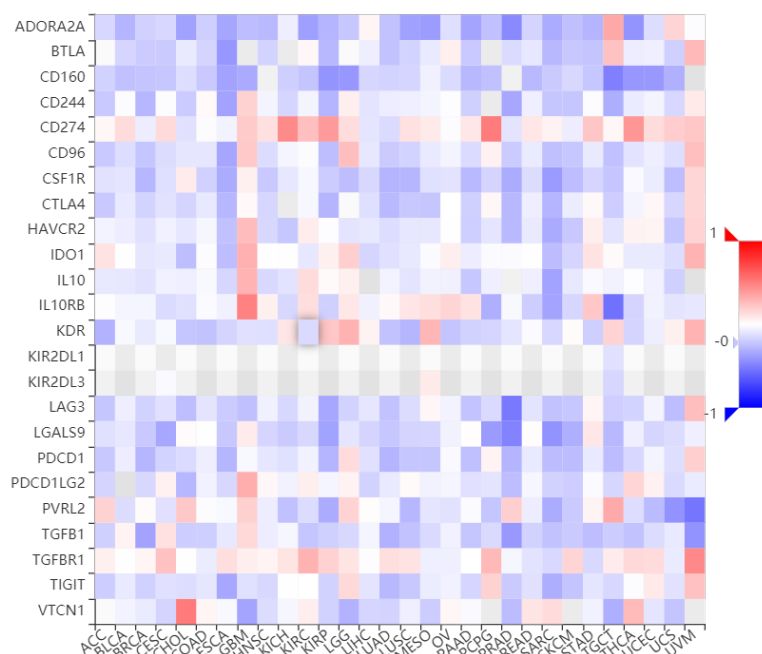**D**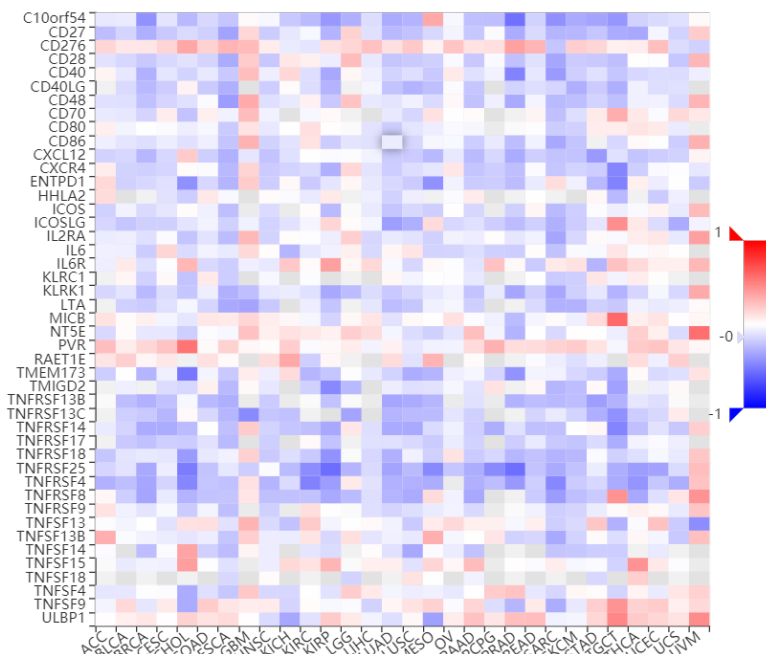**E**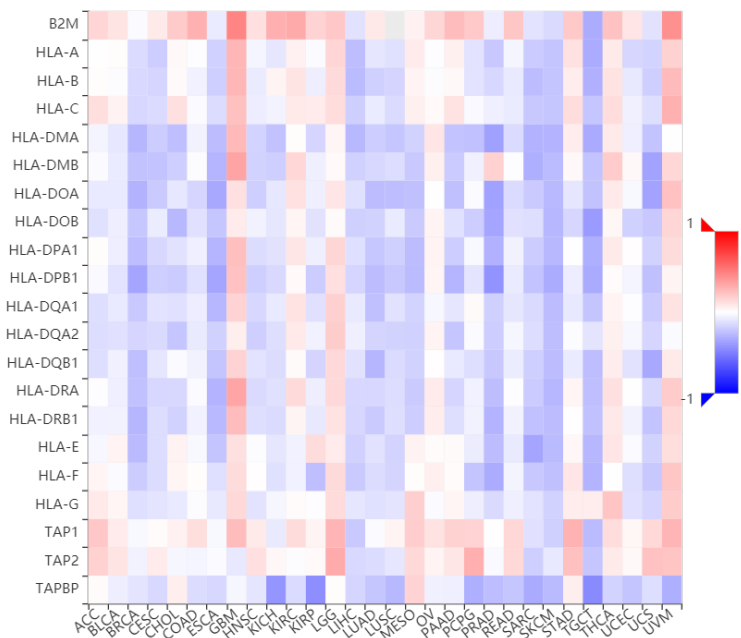

Supplement: Supplementary Figure 7 — Spearman correlation heatmap showing the relationship between FBXO22 expression and, three kinds of immunomodulators, and chemokines. Spearman correlations between expression of FBXO22 and (A) chemokines, (B) receptors, (C) immunoinhibitors, (D) immunostimulators, and (E) major histocompatibility complex components across human cancers. [file Image_7.pdf]
