# Supplementary material for: Pan-Cancer Analyses Reveal Oncogenic Role and Prognostic Value of F-Box Only Protein 22
Source: Front Oncol. 2022 Jan 24;11:790912. doi: 10.3389/fonc.2021.790912 (PMC8818750; doi:10.3389/fonc.2021.790912)
Supplement: Supplementary Figure 6 — Spearman correlation heatmap showing the relationship between abundance of tumor-infiltrating lymphocytes (TILs) and FBXO22 expression, copy number variation (CNV), and methylation in TISIDB database. Spearman correlations between (A) FBXO22 expression, (B) FBXO22 CNV, and (C) FBXO22 methylation and TIL abundance. [file Image_6.pdf]

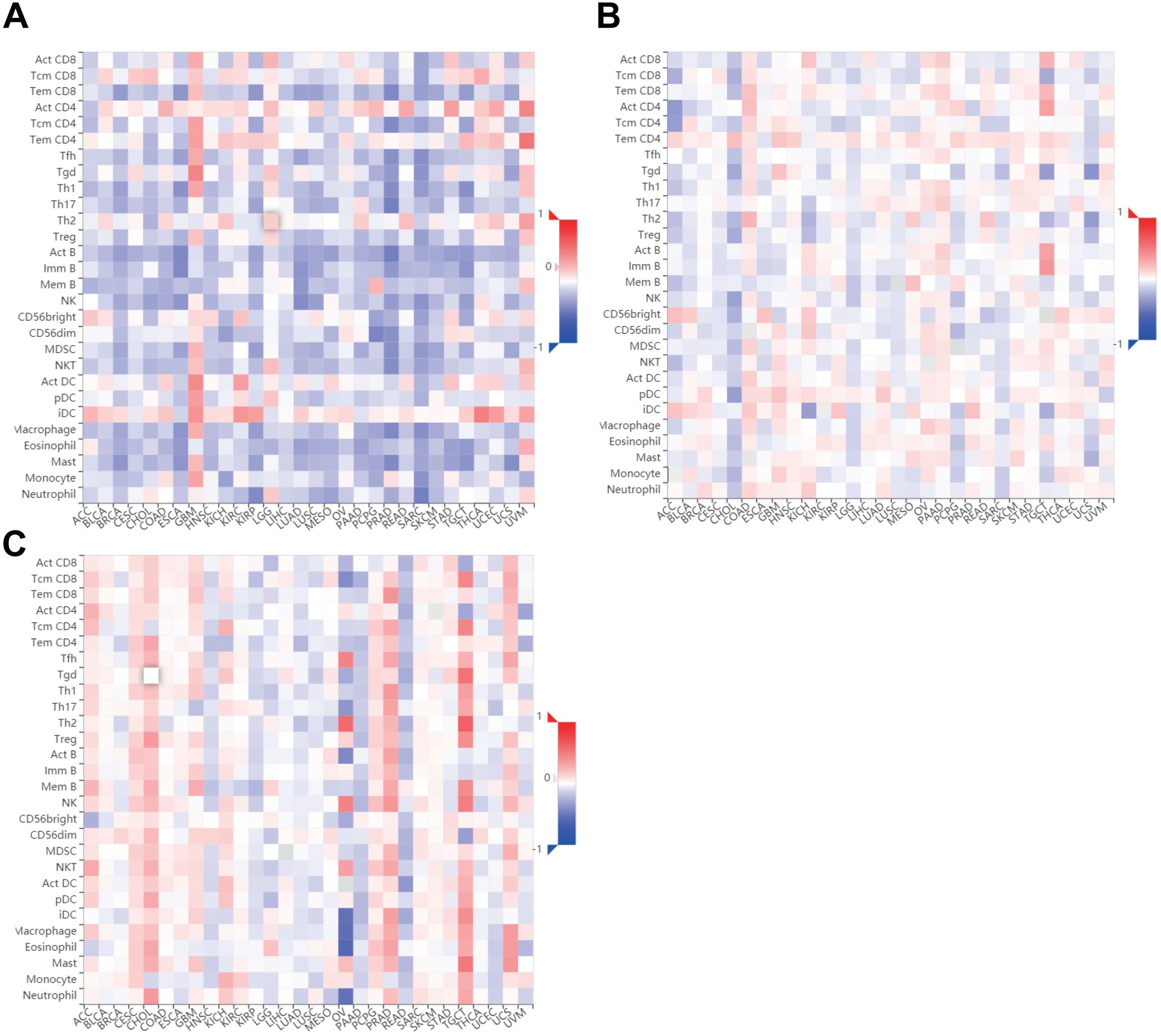
A
B
C
Act CD8
Tcm CD8
Tem CD8
Act CD4
Tcm CD4
Tem CD4
Tfh
Tgd
Th1
Th17
Th2
Treg
Act B
Imm B
Mem B
NK
CD56bright
CD56dim
MDSC
NKT
Act DC
pDC
iDC
Macrophage
Eosinophil
Mast
Monocyte
Neutrophil
ACC BLCA BRCA CESC CHOL COAD ESCA GBM HNSC KICH KIRC KIRP LGG LIHC LUAD LUSC MESO OV PAAD PCPG PRAD READ SARC SKCM STAD TGCT THCA UCEC UCS UVM
1
0
-1
