# Supplementary material for: Pan-Cancer Analyses Reveal Oncogenic Role and Prognostic Value of F-Box Only Protein 22
Source: Front Oncol. 2022 Jan 24;11:790912. doi: 10.3389/fonc.2021.790912 (PMC8818750; doi:10.3389/fonc.2021.790912)
Supplement: Supplementary Figure 8 — Spearman correlation heatmap showing the relationship between FBXO22 expression and tumor infiltrating leukocytes (TILs) based on different algorithms. (A) CIBERSORT-ABS, (B) CIBERSORT, (C) EPIC, (D) TIMER, (E) MCP-counter, (F) quantTIseq, (G) xCell, and (H) TIP. [file Image_8.pdf]

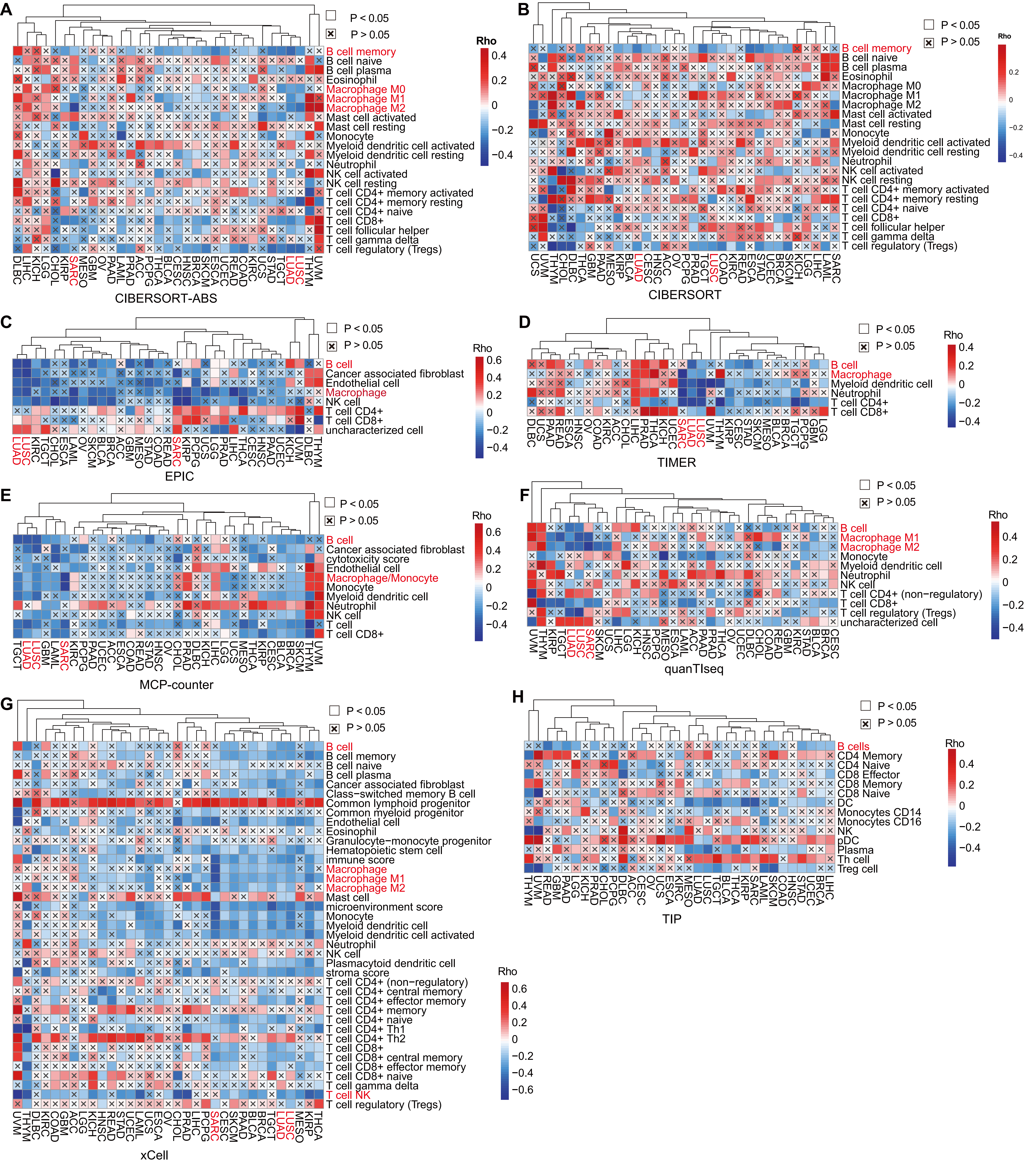
A
P < 0.05
P > 0.05
Rho
0.4
0.2
0
−0.2
−0.4
B cell memory
B cell naive
B cell plasma
Eosinophil
Macrophage M0
Macrophage M1
Macrophage M2
Mast cell activated
Mast cell resting
Monocyte
Myeloid dendritic cell activated
Myeloid dendritic cell resting
Neutrophil
NK cell activated
NK cell resting
T cell CD4+ memory activated
T cell CD4+ memory resting
T cell CD4+ naive
T cell CD8+
T cell follicular helper
T cell gamma delta
T cell regulatory (Tregs)
DLBC LIHC KICH LGG CHOL KIRP SARC MESO GBM OV PAAD LAML ACC PCPG THCA BLCA CESC HNSC BRCA SKCM ESCA UCEC READ COAD KIRC UCS STAD TGCT LUAD LUSC THYM UVM
CIBERSORT-ABS
B
P < 0.05
P > 0.05
Rho
0.2
0
−0.2
−0.4
B cell memory
B cell naive
B cell plasma
Eosinophil
Macrophage M0
Macrophage M1
Macrophage M2
Mast cell activated
Mast cell resting
Monocyte
Myeloid dendritic cell activated
Myeloid dendritic cell resting
Neutrophil
NK cell activated
NK cell resting
T cell CD4+ memory activated
T cell CD4+ memory resting
T cell CD4+ naive
T cell CD8+
T cell follicular helper
T cell gamma delta
T cell regulatory (Tregs)
UCS UVM THYM CHOL DLBC THCA GBM PAAD MESO KIRP BLCA LUAD HNSC CESC ACC OV PCPG PRAD TGCT LUSC COAD KIRC READ ESCA STAD UCEC BRCA SKCM KICH LGG LIHC LAML SARC
CIBERSORT
C
P < 0.05
P > 0.05
Rho
0.6
0.4
0.2
0
−0.2
−0.4
B cell
Cancer associated fibroblast
Endothelial cell
Macrophage
NK cell
T cell CD4+
T cell CD8+
uncharacterized cell
LUAD LUSC KIRC TGCT CHOL ESCA LAML OV SKCM BLCA BRCA ACC GBM MESO STAD COAD READ SARC KIRP PCPG UCS LGG PRAD LIHC THCA CESC HNSC PAAD UCEC KICH UVM DLBC THYM
EPIC
D
P < 0.05
P > 0.05
Rho
0.4
0.2
0
−0.2
−0.4
B cell
Macrophage
Myeloid dendritic cell
Neutrophil
T cell CD4+
T cell CD8+
DLBC UCS PAAD READ ESCA HNSC OV COAD KIRC ACC CHOL LIHC PRAD THCA KICH UCEC SARC LUSC LUAD UVM THYM KIRP CESC STAD SKCM MESO BLCA BRCA TGCT PCPG GBM LGG
TIMER
E
P < 0.05
P > 0.05
Rho
0.6
0.4
0.2
0
−0.2
−0.4
B cell
Cancer associated fibroblast
cytotoxicity score
Endothelial cell
Macrophage/Monocyte
Monocyte
Myeloid dendritic cell
Neutrophil
NK cell
T cell
T cell CD8+
TGCT LUAD LUSC LAML GBM KIRC SARC PCPG PAAD UCEC ESCA COAD READ STAD HNSC OV CHOL PRAD DLBC KICH LIHC LGG UCS MESO THCA KIRP CESC BLCA BRCA SKCM THYM UVM
MCP-counter
F
P < 0.05
P > 0.05
Rho
0.4
0.2
0
−0.2
−0.4
B cell
Macrophage M1
Macrophage M2
Monocyte
Myeloid dendritic cell
Neutrophil
NK cell
T cell CD4+ (non−regulatory)
T cell CD8+
T cell regulatory (Tregs)
uncharacterized cell
UVM THYM KIRP TGCT LUAD LUSC SARC SKCM UCS LIHC LGG KICH HNSC PCPG MESO ESCA LAML ACC PAAD PRAD THCA OV UCEC DLBC CHOL COAD READ GBM KIRC STAD BLCA BRCA CESC
quanTIseq
G
P < 0.05
P > 0.05
Rho
0.6
0.4
0.2
0
−0.2
−0.4
−0.6
B cell
B cell memory
B cell naive
B cell plasma
Cancer associated fibroblast
Class−switched memory B cell
Common lymphoid progenitor
Common myeloid progenitor
Endothelial cell
Eosinophil
Granulocyte−monocyte progenitor
Hematopoietic stem cell
immune score
Macrophage
Macrophage M1
Macrophage M2
Mast cell
microenvironment score
Monocyte
Myeloid dendritic cell
Myeloid dendritic cell activated
Neutrophil
NK cell
Plasmacytoid dendritic cell
stroma score
T cell CD4+ (non−regulatory)
T cell CD4+ central memory
T cell CD4+ effector memory
T cell CD4+ memory
T cell CD4+ naive
T cell CD4+ Th1
T cell CD4+ Th2
T cell CD8+
T cell CD8+ central memory
T cell CD8+ effector memory
T cell CD8+ naive
T cell gamma delta
T cell NK
T cell regulatory (Tregs)
UVM THYM DLBC KIRC COAD GBM ACC LGG KICH HNSC READ STAD UCEC LAML UCS ESCA OV CHOL PRAD LIHC PCPG SARC CESC SKCM PAAD BLCA BRCA TGCT LUAD LUSC MESO KIRP THCA
xCell
H
P < 0.05
P > 0.05
Rho
0.4
0.2
0
−0.2
−0.4
B cells
CD4 Memory
CD4 Naive
CD8 Effector
CD8 Memory
CD8 Naive
DC
Monocytes CD14
Monocytes CD16
NK
pDC
Plasma
Th cell
Treg cell
THYM UVM READ GBM PAAD LGG KICH PRAD CHOL PCPG DLBC ACC CESC OV UCS ESCA KIRC MESO LUAD LUSC TGCT BLCA THCA KIRP SARC LAML SKCM COAD HNSC STAD UCEC BRCA LIHC
TIP
